# Refined definition of the critical micelle concentration and application to alkyl maltosides used in membrane protein research†

**DOI:** 10.1039/d2ra07440k

**Published:** 2023-03-22

**Authors:** Adrian Bothe, Athina Zouni, Frank Müh

**Affiliations:** a Institut für Biologie, Humboldt Universität zu Berlin Leonor-Michaelis-Haus, Philippstrasse 13 D-10095 Berlin Germany; b Institut für Theoretische Physik, Johannes Kepler Universität Linz Altenberger Strasse 69 A-4040 Linz Austria frank.mueh@jku.at

## Abstract

The critical micelle concentration (CMC) of nonionic detergents is defined as the breaking point in the monomer concentration as a function of the total detergent concentration, identified by setting the third derivate of this function to zero. Combined with a mass action model for micelle formation, this definition yields analytic formulae for the concentration ratio of monomers to total detergent at the CMC and the relationship between the CMC and the free energy of micellization *g*_mic_. The theoretical breaking point is shown to coincide with the breaking point of the experimental titration curve, if the fluorescence enhancement of 8-anilino-1-naphthalene-sulfonic acid (ANS) or a similar probe dye is used to monitor micelle formation. Application to a series of *n*-alkyl-β-d-maltosides with the number of carbon atoms in the alkyl chain ranging from 8 to 12 demonstrates the good performance of a molecular thermodynamic model, in which the free energy of micellization is given by *g*_mic_ = *σΦ* + *g*_pack_ + *g*_st_. In this model, *σ* is a fit parameter with the dimension of surface tension, *Φ* represents the change in area of hydrophobic molecular surfaces in contact with the aqueous phase, and *g*_pack_ and *g*_st_ are contributions, respectively, from alkyl chain packing in the micelle interior and steric repulsion of detergent head groups. The analysis of experimental data from different sources shows that varying experimental conditions such as co-solutes in the aqueous phase can be accounted for by adapting only *σ*, if the co-solutes do not bind to the detergent to an appreciable extent. The model is considered a good compromise between theory and practicability to be applied in the context of *in vitro* investigations of membrane proteins.

## Abstract 2

A refined definition of the critical micelle concentration is connected to fluorescence methods and applied to test a molecular thermodynamic model of nonionic detergents used for *in vitro* studies of membrane proteins.

## Graphical abstract

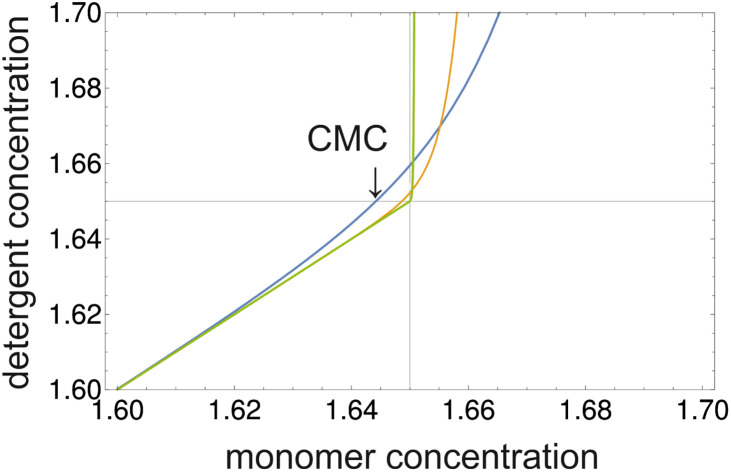

## 1 Introduction

Detergents (or surfactants) are amphiphilic substances with major contributions to many aspects of modern life and play an essential role in scientific applications such as membrane protein research.^1–3^ Detergent molecules consist of a hydrophilic (polar) segment and a hydrophobic part, covalently joined together.^4^ The hydrophobic section is often made up of a hydrocarbon chain, i. e. an *n*-alkyl group. In an aqueous phase, detergents tend to form globular aggregates termed micelles, where the hydrophobic parts of individual detergent molecules are located in the center of the aggregate and shielded from the surrounding water by the polar groups at the surface of the micelle. Mild uncharged detergents like alkyl maltosides can be used to extract membrane proteins from native biological membranes and solubilize them in an aqueous environment by binding of detergent molecules to hydrophobic domains and formation of a detergent belt surrounding those parts of the protein.^5^ The protein plus detergent belt is referred to as the protein-detergent complex (PDC), and it is soluble in water due to its polar exterior. Only after such solubilization with detergents are membrane proteins accessible with structural biology methods such as cryo-electron microscopy (cryo-EM)^1^ or X-ray diffraction (XRD).^2^ The structure determination in turn is crucial to the functional understanding of proteins and enzymes. A plethora of essential proteins is embedded into membranes, which is why detergents are indispensable to the study of many fundamental biological processes such as respiration and photosynthesis.

However, the use of detergents poses additional challenges to these methods. In cryo-EM, the presence of free detergent micelles along with PDCs affects the classification of projections, if the micelles are similar to the PDC in size and shape or due to interactions between both types of particles.^1^ For XRD, the growth of protein crystals is a necessary prerequisite, and finding suitable crystallizing conditions is challenging.^6^ When crystallizing intrinsic membrane proteins, special attention has to be paid to the detergent belt, its stability and possible interactions between the detergent and the buffer components (*e.g.*, precipitants, additives), because the detergent may interfere with attractive intermolecular forces that hold proteins together within the crystal lattice, or the detergent belt may be destabilized by components of the buffer. In the particular case of photosystem II of oxygenic photosynthesis, various types of crystals with different quality are obtained depending on detergent type and concentration.^6,7^ Accordingly, much effort was put recently into the characterization of the detergent belt in PDCs employing small-angle scattering methods.^8–10^ A detailed characterization of the detergent's self-aggregation behavior is clearly beneficial to understanding the outcomes of those experiments as well as the growth of membrane protein crystals.

The behavior of detergents in aqueous solutions is mainly governed by interactions between the detergent molecule and the solvent (water), as well as among detergent molecules. These interactions are a direct function of the detergent's molecular structure, which in a molecular thermodynamic model can be linked to the free energy difference between the detergent monomer and the micelle.^3,11–16^ A quantity of particular interest is the critical micelle concentration (CMC), which is the total detergent concentration that must be exceeded for micellar structures to be formed in the solution^11–14^ and for hydrophobic substances or membrane proteins to be solubilized.^17^

In our earlier work,^3^ we investigated the influence of poly(ethylene glycol) (PEG), a common precipitant used to induce protein crystallization, on the CMC of alkyl maltosides in order to better understand the detergent behavior under the conditions of membrane protein crystallization. The CMC was measured by employing fluorescence techniques involving the dye 8-anilino-1-naphthalene-sulfonic acid (ANS), which is known to increase its fluorescence intensity upon binding to micelles.^18,19^ The micelle formation was modelled with molecular thermodynamics,^14^ which was modified to explore the role of surface tension in the description of hydrophobic molecular surfaces in contact with water. Although data exist for PEG types of different molecular weight, the original analysis^3^ was restricted to PEG2000. In the course of analyzing the remaining data, we realized that there is room for improvement of the molecular thermodynamic model for the micelle formation of alkyl maltosides even in the absence of PEG. In particular, the modelling of the micellization free energy in terms of hydrophobic molecular surfaces and an associated surface tension required more care in a biophysical context, where the aqueous solutions contain additives like salts and buffer. Eventually, the modelling reached a status, where it became necessary to reconsider the definition of the CMC in the context of its experimental determination by using fluorescence probes. The resulting values for the surface tension were confronted with literature data, which ultimately led to an analysis of the underlying thermodynamics and common approximations to uncover systematic errors.

The goal of the present paper is to describe the improvement of the model of micelle formation for alkyl maltosides alongside a fundamental discussion of the underlying thermodynamics and a refined definition of the CMC motivated by the classical work of Philipps.^20^ This analysis will set the stage for future work to apply the modelling philosophy to analyze the influence of PEG and to describe the formation of PDCs in detergent solutions.

## 2 Theoretical basis and computational methods

### 2.1 Thermodynamics of micelle formation

The Gibbs free energy *G* of a composite system consisting of *N*_*j*_ particles of type *j* with chemical potential *μ*_*j*_ is given by the Euler equation:^21,22^1
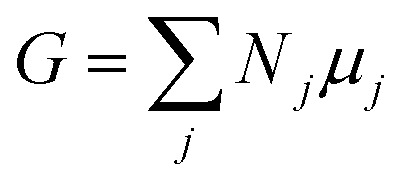


To describe an aqueous solution with *N*_wat_ water molecules containing besides *N*_det_ detergent molecules a total of *N*_cos_ molecules of co-solutes, we model *G* as consisting of two parts:^23–26^

2 *G* = *G*_f_ + *G*_mix_

where we neglect interactions between solutes (ideal solution). The free energy of formation *G*_f_ is given by3
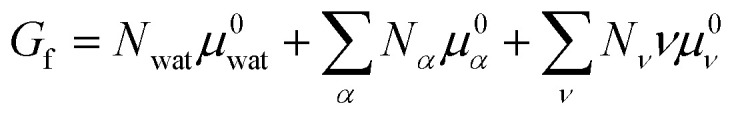
Here, *μ*^0^_i_ is the standard chemical potential of species i. The index *α* counts the species of co-solutes, while the index *ν* refers to a detergent aggregate with aggregation number *ν*. Hence, the total number of co-solute molecules is given by4
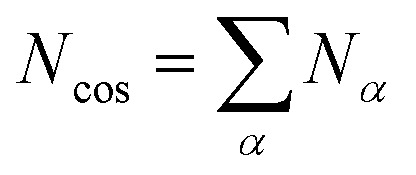
while the total number of detergent molecules is5
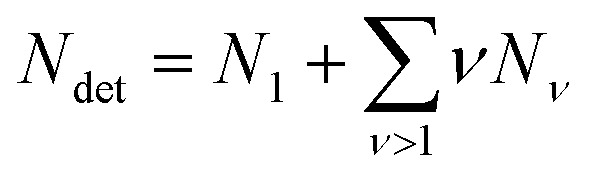
With the exception of water, for any species i, *μ*^0^_i_ can be interpreted as the change in Gibbs free energy of the solution when a single molecule of type i is added to the solution without considering mixing effects.^23^ (Actually, *μ*^0^_i_ represents a Henry's law standard state, where the behavior of a sufficiently diluted solution is extrapolated to *X*_i_ = 1.^27,28^) Note that the standard chemical potential for a micelle of size *ν* is *νμ*^0^_*ν*_ and contains the interaction between detergent molecules within the micelle. (Thus, *μ*^0^_*ν*_ is the standard chemical potential of a micelle of size *ν* per detergent molecule in the micelle.) In contrast, *μ*^0^_wat_ is the free energy change when a water molecule is added to pure water and accounts for water–water interactions.^23^ (Thus, *μ*^0^_wat_ represents a Raoult's law standard state for *X*_wat_ = 1.^27,28^) With the total number of particles *N*_tot_ = *N*_wat_ + *N*_det_ + *N*_cos_, we define for water and co-solutes the mole fractions as *X*_i_ = *N*_i_/*N*_tot_, whereas for the detergent, we define6
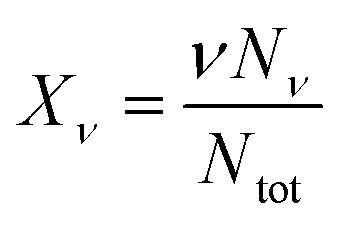
so that *X*_*ν*_ is the mole fraction of detergent in micelles of size *ν* (rather than the mole fraction of micelles of size *ν*). With these definitions, the free energy of mixing in the ideal solution model^26^ has the form7
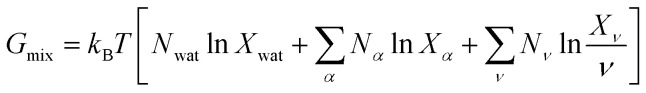
where *T* is the absolute temperature and *k*_B_ Boltzmann's constant. Note that −*G*_mix_/*T* reflects the entropy of mixing.^23,26^

To find the size distribution of micelles at equilibrium, we have to determine the minimum of *G* with respect to a variation of the numbers *N*_*ν*_ subject to the constraint stated in eqn (5). We apply the method of Lagrange multipliers by defining a function8
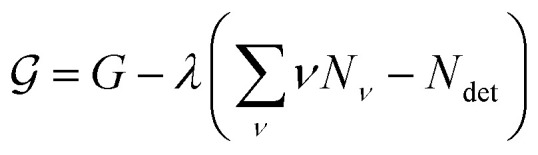
with the Lagrange multiplier *λ* and setting 
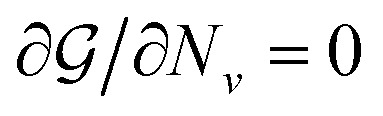
 for all *ν*. This procedure yields the equilibrium condition9
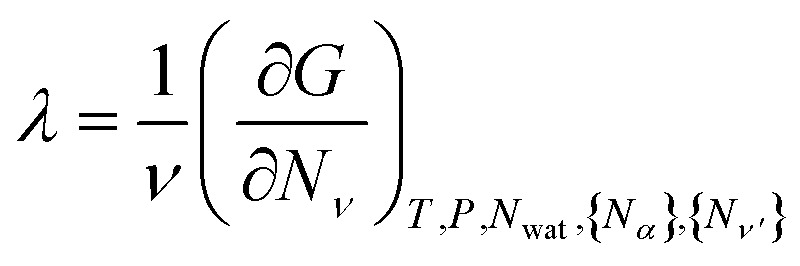


From eqn (9), we can identify *λ* as the chemical potential of detergent monomers *μ*_1_, which has to be equal to the chemical potential of detergent molecules in micelles of size *ν*. The resulting equation^11^10
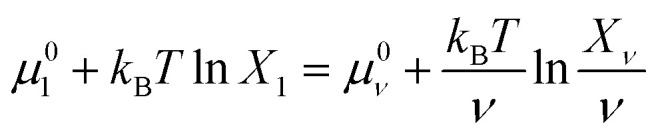
can be rearranged to yield the micellar size distribution11
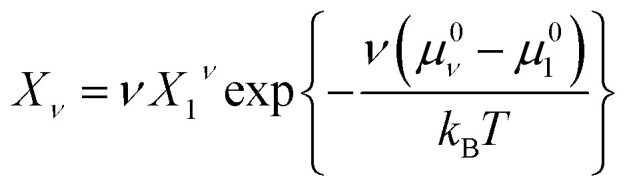


Note that this size distribution function assumes ideal mixing and is restricted to dilute solutions where interactions between micelles can be ignored.^11,26^

At this point, we make the simplifying assumption that the micellar size distribution is sufficiently narrow, so that it is adequate to consider only one type of micelle with a fixed aggregation number *ν* = *m*. We expect this to be a good approximation for alkyl maltosides at concentrations well above the CMC.^3,29,30^ (The validity of this assumption in the vicinity of the CMC is further discussed in Section 4.2.) For this particular aggregation number, we introduce the abbreviation12
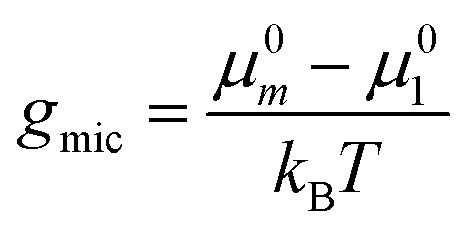
so that the mole fraction of detergent in micelles becomes

13 *X*_mic_ ≡ *X*_*m*_ = *mX*_1_^*m*^ e^−*mg*_mic_^

Eqn (13) can be used to define a critical monomer concentration (*X*_1_)_crit_ that is usually taken as the CMC following an argumentation given by Israelachvili:^11^ Since *X*_*m*_ is a mole fraction, it can never exceed unity. Consequently, the monomer mole fraction *X*_1_ must not exceed some critical value that is given by:14
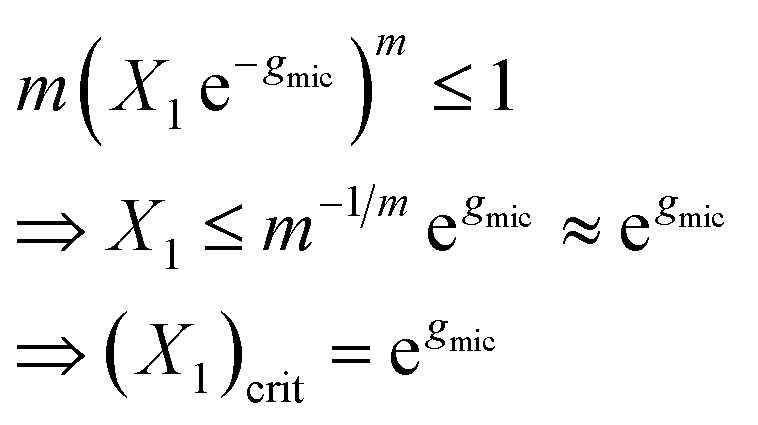


Note that *m*^−1/*m*^ → 1 for sufficiently large *m*. In eqn (14), (*X*_1_)_crit_ is the critical mole fraction of detergent monomers. However, since it is assumed that there is no significant amount of micelles present below (*X*_1_)_crit_, it is usually identified with the total mole fraction of detergent and employed as an approximation for the CMC.^11–13^ In the present work, we use a refined definition of the CMC (see Section 2.2) in order to better describe experimental data. Irrespectively, the relationship between the CMC and the total mole fraction of detergent at the CMC, *X*_CMC_, is given by

15 CMC = *c*_tot_*X*_CMC_

where the total molarity of the solution, *c*_tot_, is described in Section 2.6.

### 2.2 Refined definition of the critical micelle concentration

**Fig. 1:**
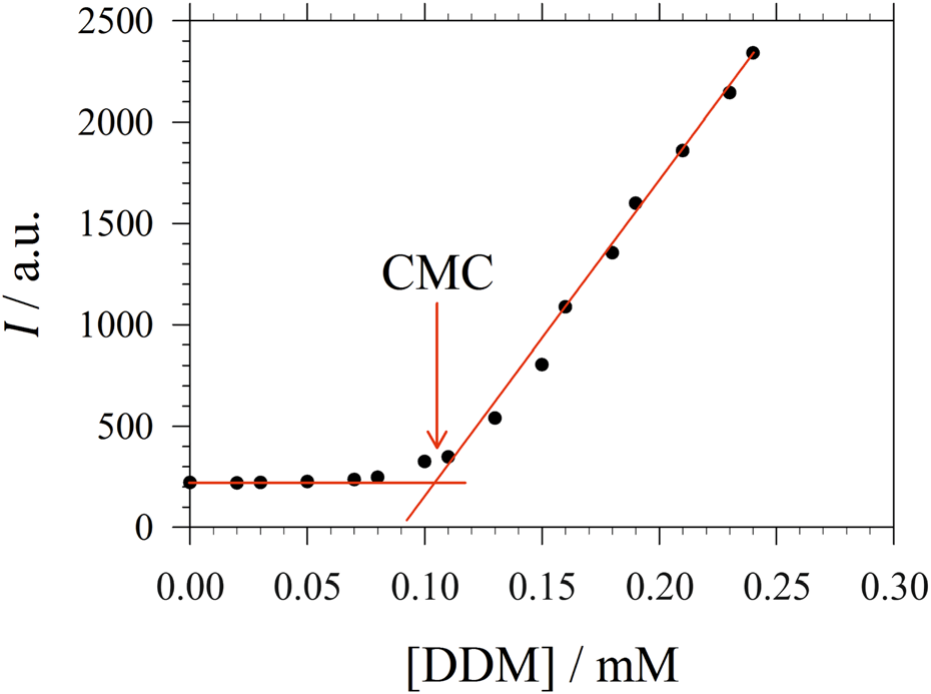
Representative titration curve of the ANS fluorescence intensity *I versus* the total detergent concentration of DDM (dodecyl maltoside) in 100 mM PIPES (pH 7.0) and 5 mM CaCl_2_ (same experimental data as in Fig. 1 of ref. 3). The straight lines illustrate the graphical procedure to determine the CMC. Figure made with SigmaPlot 13 (© 2014 Systat Software Inc.).

For the sake of simplifying the notation, we introduce the following abbreviations: the total mole fraction of detergent is *x*: = *N*_det_/*N*, the mole fraction of detergent monomers is *y*: = *X*_1_, and the mole fraction of detergent in micelles (with aggregation number *m*) is *z*: = *X*_mic_ = *my*^*m*^ e^−*mg*_mic_^. Then, the mass balance of detergent is given by *x* = *y* + *z*. To determine the CMC experimentally, a suitable observable *ϕ* (*e.g.*, the fluorescence intensity of ANS) is monitored as a function of *x*. Usually, *ϕ*(*x*) exhibits a sharp breaking point that can be used to read off the CMC (see Fig. 1 for a representative titration curve). For a better definition of the breaking point, Phillips proposed the condition^20^16
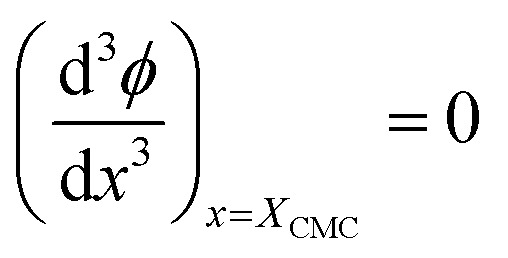
which we will refer to as the *ϕ*-condition. However, the relationship between *ϕ* and *y* is in general complex or unknown, and by applying the *ϕ*-condition, a variety of CMC values might be obtained depending on the experimental methods used. For a self-consistent definition of the CMC that is independent of the experimental method, Al-Soufi *et al.*^31^ proposed instead17
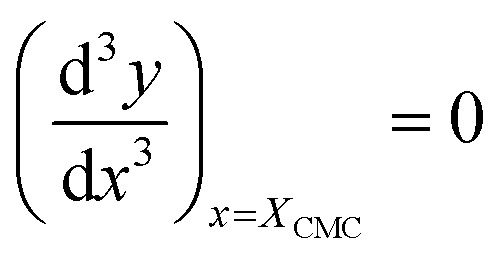
which we term the *y*-condition.

In the following, a prime indicates a derivative with respect to *x*, while a dot denotes a derivative with respect to *y*. Then, in order to exploit the *y*-condition, we define the function *f*(*x*,*y*) = *x* − *y* − *z*(*y*) = 0 and obtain from implicit differentiation:18
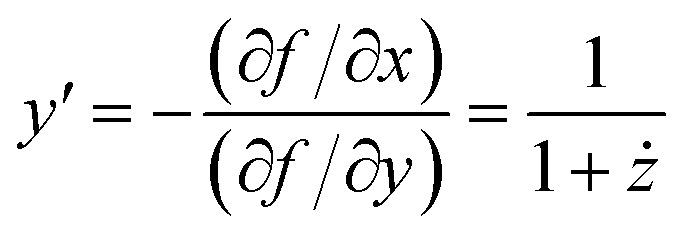


From eqn (18), we obtain the second and third derivative with respect to *x* and finally find that eqn (17) implies19
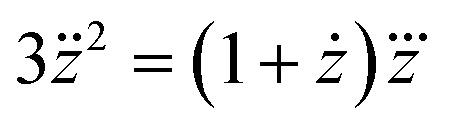


We note that it follows from the mass balance of detergent that *z*′ = 1 − *y*′, *z*′′ = −*y*′′, and *z*′′′ = −*y*′′′, so that the *y*-condition can also be expressed as *z*′′′ = 0, *i.e.*, in terms of a breaking point in the concentration of detergent in micelles as a function of total detergent concentration.

**Fig. 2:**
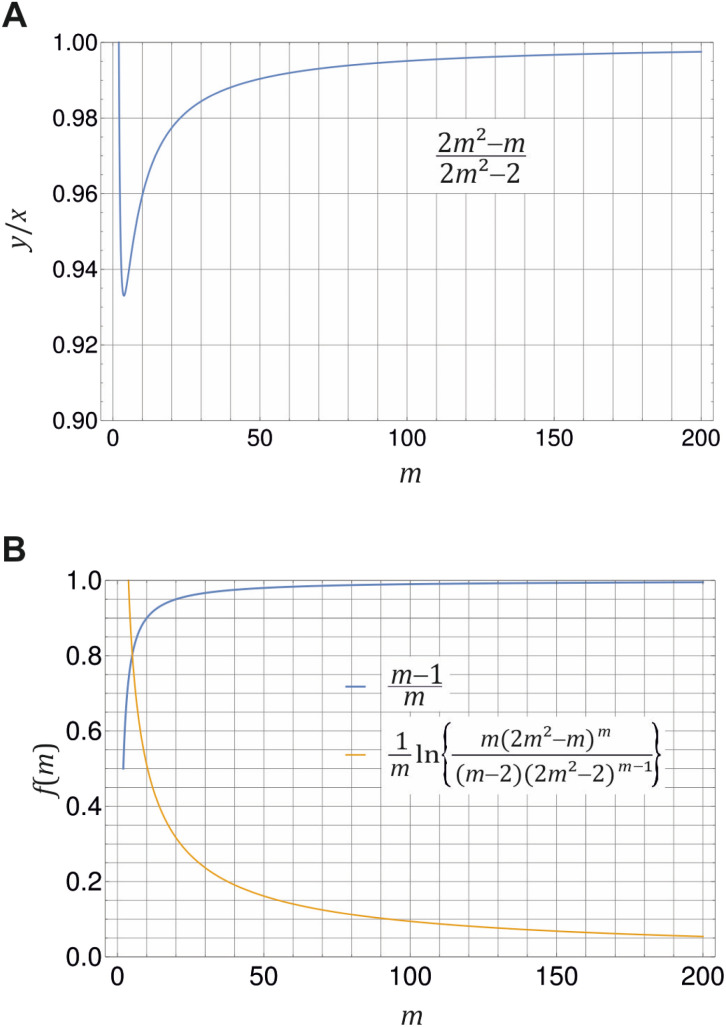
Illustration of eqn (20) (A) and (21) (B) for different values of *m* ≥ 2. (A) Ratio of monomer to total detergent concentration (*y*/*x*) at the breaking point as a function of aggregation number *m* according to eqn (20). (B) The prefactor of ln *X*_CMC_ and the second term in eqn (21) as a function of *m*. Plots made with Mathematica 11.2 (© 1988–2017 Wolfram Research).

**Fig. 3:**
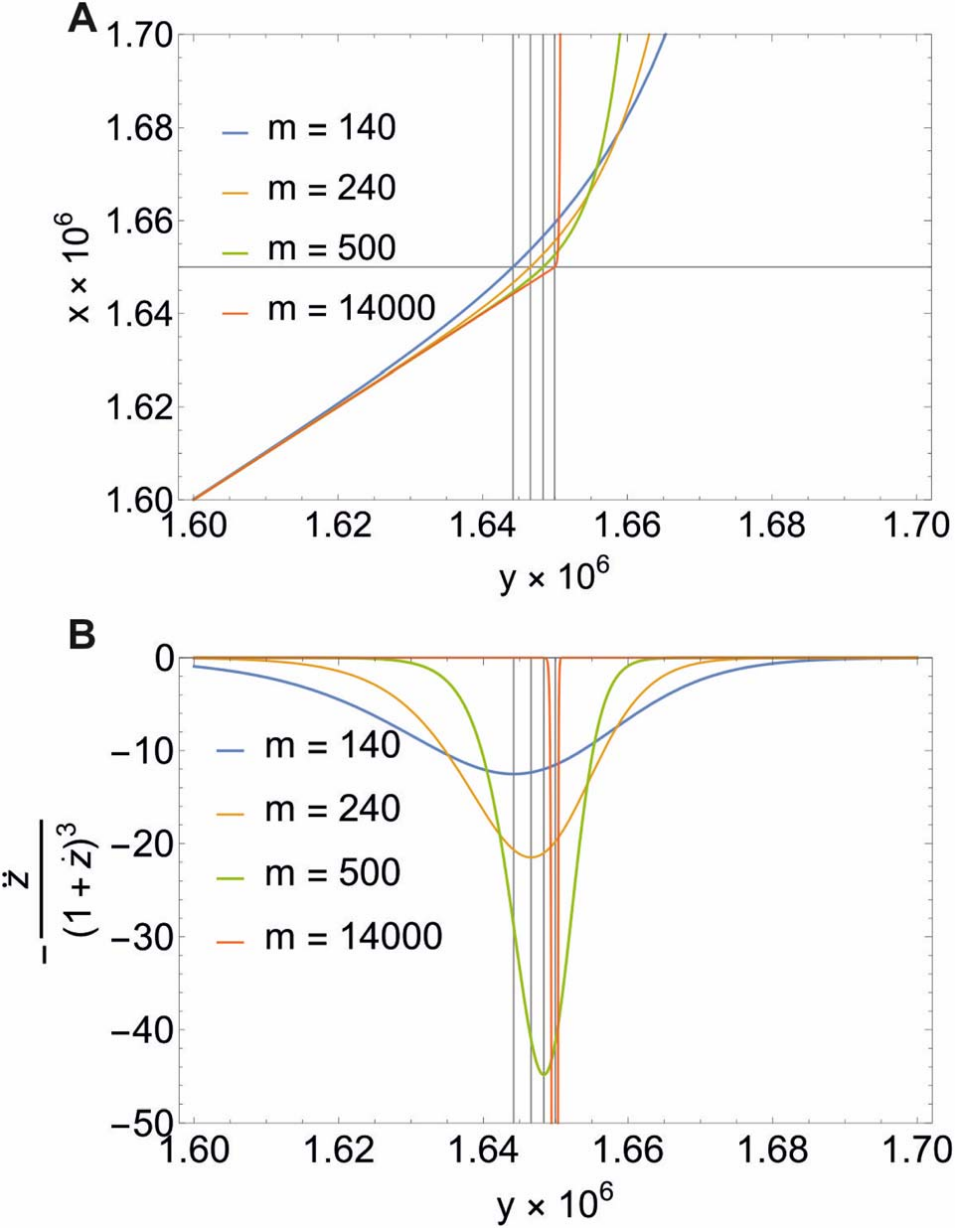
Illustration of eqn (22) (A) and the second derivative 
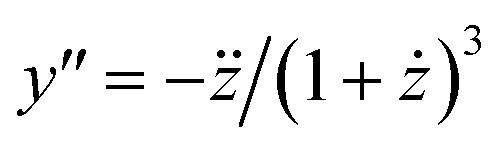
 (B) for different values of *m* and *X*_CMC_ = 1.65 × 10^−6^ (DDM). Note that all concentrations are in mole fraction units. The horizontal line in A indicates the value of *X*_CMC_. The vertical lines in (A) and (B) are the corresponding values for *y* at the breaking point according to eqn (20). Figure made with Mathematica 11.2 (© 1988–2017 Wolfram Research).

Combining eqn (19) with eqn (13), we find that20
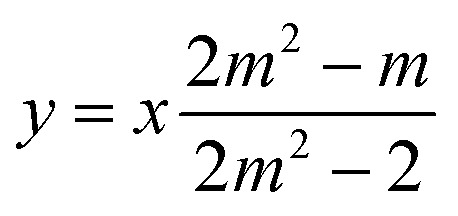
at the breaking point. The resulting ratio of monomer to total detergent concentration at the breaking point, *y*/*x*, as a function of the aggregation number *m* is illustrated in Fig. 2A. This ratio approaches unity only in the limit *m* → ∞ and remains smaller than 1 for finite *m* > 2. This behavior indicates that a certain amount of detergent is bound in micelles at the breaking point. With increasing *m*, the break in the (theoretical) titration curve becomes sharper (*cf.*Fig. 3A) and less and less detergent is bound in micelles at the breaking point. In the limit *m* → ∞, which corresponds to the traditional definition of the CMC, *y*/*x* = 1 at the breaking point, and the monomer concentration equals the total detergent concentration right before the onset of micelle formation as is usually presumed.

If we define *x* at the breaking point as *X*_CMC_, we can relate the CMC to the free energy of micellization according to21
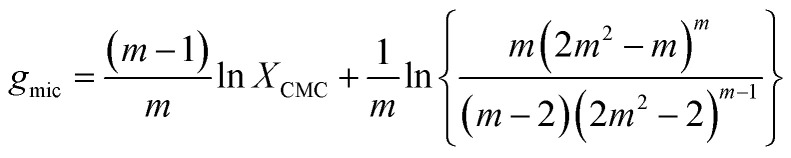
In the limit of large *m*, we recover the well-known approximate relationship *g*_mic_ ≈ ln *X*_CMC_. It is instructive to see, how the corrections to the latter approximation change with *m* (Fig. 2B). The prefactor of ln *X*_CMC_ in eqn (21) approaches unity quite fast, so that it is of minor importance for aggregation numbers around 100. However, in the same range of aggregation numbers, the additional second term in eqn (21) is still in the order of 0.1 and not necessarily negligible.

The mass balance for the detergent now reads22
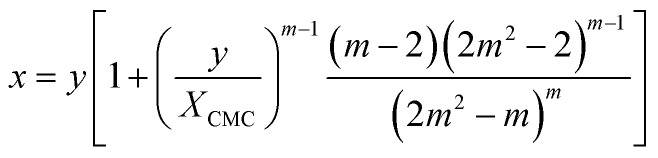


Illustrative theoretical titration curves are shown in Fig. 3A. Note that these curves are inverted, *i.e.*, shown is *x*(*y*) for *X*_CMC_ = 1.65 × 10^−6^ (corresponding to DDM) and aggregation numbers of 140 (the real one for DDM) as well as 240, 500, and 14 000. It can be seen that the curves become sharper and the monomer concentration above the breaking point becomes smaller with increasing *m* (keeping *X*_CMC_ constant). The largest value of *m* is intended to approximate the limit *m* → ∞ that reflects the traditional definition of the CMC. Apparently, in this limit, the monomer concentration above the CMC is underestimated.

It is not so obvious from the curves in Fig. 3A that the breaking point is indeed at *X*_CMC_ = 1.65 × 10^−6^ (except, possibly, for *m* = 14 000). To check the consistency of the formalism, we computed the second derivative 
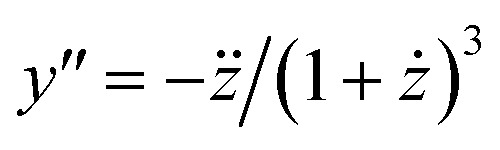
, which is an intermediate step in the derivation of eqn (19). This function should exhibit a minimum for the value of *y* at the breaking point, which is related to *x* = *X*_CMC_*via*eqn (20). As can be seen from Fig. 3B, this is indeed the case. However, the curve for *m* = 14 000 is not completely shown in Fig. 3B. It has a sharp minimum close to *y* = *X*_CMC_ = 1.65 × 10^−6^ as expected in the limit *m* → ∞.

It should be noted that eqn (18)–(22) are, to the best of our knowledge, new results that have not been published before.

### 2.3 Experimental reference data and interpretation of titration curves

A significant increase in the fluorescence intensity *I* above the CMC is known for the indicator dye ANS.^18,19^ In this case, the CMC is defined as the total detergent concentration at which the slope of the fluorescence intensity abruptly increases (see Fig. 1). This definition corresponds to the *ϕ*-condition with *ϕ* = *I*. In the following, we show that in this particular case, the *ϕ*-condition coincides with the *y*-condition.

We make the simplifying assumption that at most one ANS molecule binds to a micelle and neglect binding of ANS to detergent monomers. Then, the association of ANS with micelles can be characterized by the equilibrium constant23
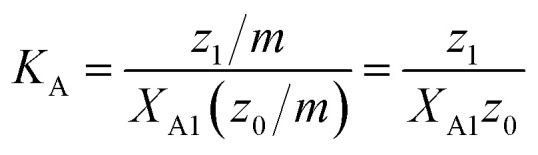
where *X*_A1_ is the mole fraction of free ANS molecules in the aqueous phase, while *z*_0_/*m* and *z*_1_/*m* are the mole fractions of micelles containing no or one ANS molecule, respectively. Under these conditions, the mass balance of detergent is *x* = *y* + *z*_0_ + *z*_1_, while that of ANS reads24
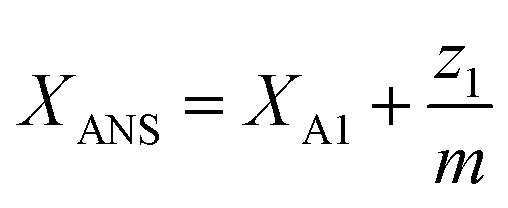
where *X*_ANS_ is the total mole fraction of ANS.

Let us denote by *I*_1_ the fluorescence intensity of one mole of ANS in an aqueous environment and by *I*_mic_ that of ANS bound to a micelle. Then, the total fluorescence intensity is25
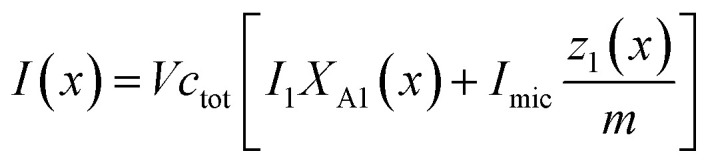
where we have indicated that *I* depends on *x via X*_A1_ and *z*_1_, and *V* is the volume of the sample. Now, from the mass balance of ANS, it follows that26
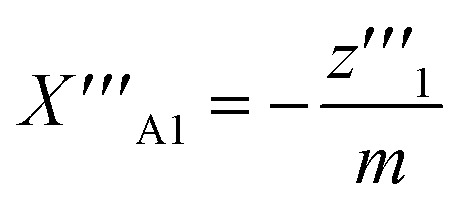
which is analogous to the relationship between *y*′′′ and *z*′′′ mentioned in Section 2.2. In the presence of ANS, however, we have27
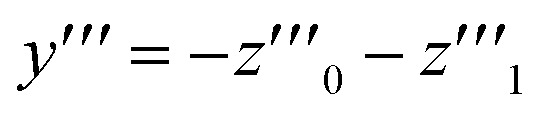


One solution for *y*′′′ = 0 under these conditions is 
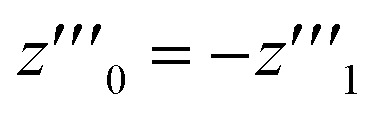
, which is, however, unphysical as it implies an unrealistically different behavior of micelles with and without ANS. A more realistic assumption is that the aggregation of *m* detergent molecules into one micelle is not significantly affected by one ANS molecule. Then, *z*_0_ and *z*_1_ should show the same dependence on *x*, which is the same as that of *z* in the absence of ANS. Hence, the *y*-condition implies 
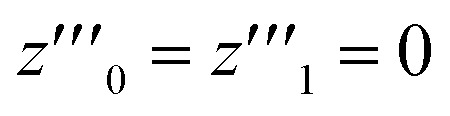
, and it follows from eqn (26) and (25) that

28 *y*′′′ = 0 ⇒ *I*′′′ = 0

The last equation means that the breaking point of the experimental curve *I*(*x*) and the theoretical curve *y*(*x*) are the same, rendering the ANS fluorescence an ideal tool to determine the CMC based on the definition given in Section 2.2.

The focus of the present work is on *n*-alkyl-β-d-maltosides C_*n*_G_2_, where “G_2_” stands for the maltose head group and “C_*n*_” for an *n*-alkyl chain with *n* carbon atoms. (Note that the “*n*” in “*n*-alkyl” stands for “normal”.) For convenience, the three detergents with *n* = 10, 11, 12 will also be referred to by their more common names DM (decyl maltoside), UDM (undecyl maltoside) and DDM (dodecyl maltoside), respectively. Our own experimental data concerning the CMC of these three detergents, which are re-analyzed here, were obtained by D. DiFiore in the course of our earlier work,^3^ measuring the fluorescence enhancement of ANS at a final concentration of 10 μM in buffered aqueous solutions containing 100 mM piperazine-1,4-bis-(2-ethanesulfonic acid) (PIPES), adjusted to pH = 7.0 with NaOH, and 5 mM CaCl_2_. The fluorescence spectra were taken with a Horiba Jobin Yvon FluoroMax-2 spectrometer. For comparison, additional experimentally determined values of the CMC and the aggregation number for alkyl maltosides with chain length *n* = 8–13 were taken from the literature.^29,30,32–45^

### 2.4 Molecular thermodynamic model of micelle formation

In the traditional molecular thermodynamic (TMT) modelling approach,^14^ the micellization free energy *g*_mic_ is decomposed into several additive contributions:

29 *g*_mic_ = *g*_tr_ + *g*_int_ + *g*_pack_ + *g*_st_

This decomposition is based on a thought process (thermodynamic cycle), in which the process of assembly of detergent molecules into a micelle is formally split into steps such as the detachment of head groups from the alkyl tails, aggregation of alkyl tails, reattachment of head groups to the alkyl tails *etc.*^46–48^ The transfer term *g*_tr_ reflects the free energy change of transferring the alkyl tail from an aqueous environment into a liquid hydrocarbon phase representing the hydrophobic core of the micelle and is calculated on the basis of experimental transfer free energies, Δ*μ*^0^_tr_, in the TMT approach.^14^ The linear dependence of *g*_tr_ on *n*, the number of carbon atoms in the alkyl chain, largely determines the well-known exponential dependence of the CMC on *n*.

The interfacial term *g*_int_ refers to the creation of a hydrocarbon–water interface due to the formation of the micellar core and is traditionally modelled as^14^30
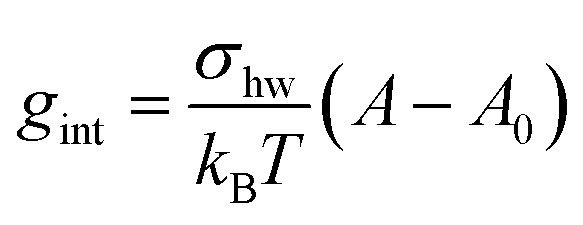
where *σ*_hw_ is the interfacial tension between the hydrophobic micellar core and the aqueous environment, *A* is the surface area of the micellar core per detergent molecule (see Section 2.5), and *A*_0_ is the area of the core per detergent molecule shielded from the aqueous phase by the sugar head group of the alkyl maltoside. In our earlier work,^3^ we followed Nagarajan and Ruckenstein^14^ and chose *σ*_hw_ as the macroscopic interfacial tension between liquid hydrocarbon (h) and water (w). In the present work, we make a different choice to be described below.

The packing term *g*_pack_ is necessary to model the energetic and entropic consequences of a different conformational distribution of the alkyl chains in the micellar core compared to a pure liquid hydrocarbon phase and is computed here in the same way as in our earlier work:^3^31
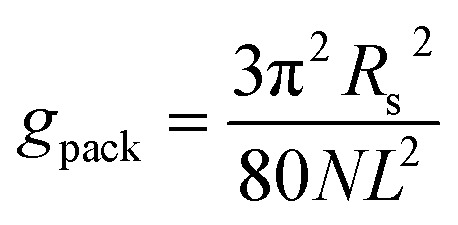


The meaning of the quantities occurring in eqn (31) and its applicability to the micelles of alkyl maltosides are discussed in Section 4.4.

Finally, the term *g*_st_ describes the steric repulsion of the head groups moving on the surface of the micellar core and is modelled by assuming a hard core repulsion interaction according to^14^32
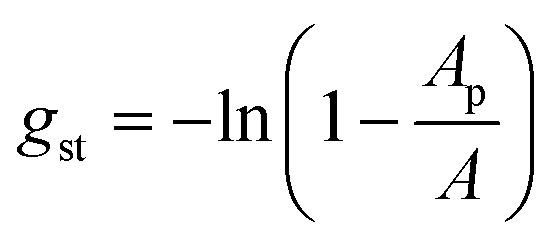
where *A*_p_ is the effective cross-sectional area of the maltose head group. Eqn (32) was said to be motivated by the van-der-Waals equation of state,^14,49^ but we were not able to find a clear-cut derivation in the literature. Therefore, a derivation is proposed in the ESI (Text S1†) that hopefully represents the intentions of the original authors.

In our earlier work,^3^ the modelling of *g*_tr_ had been altered in favor of a surface-based description. The assembly of detergent molecules implies that the area *S* of the hydrophobic surface of the alkyl tail is no longer in contact with water in the micelle except for the small part that contributes to the area *A* − *A*_0_ of the micellar core that is not shielded by the head groups; *cf.*eqn (30). Thus, it is possible to understand the transfer and interfacial terms jointly as the contribution to *g*_mic_ that is due to a change of the effective molecular surface of hydrocarbons exposed to water. In this approach, *g*_tr_ is supposed to be proportional to the surface area *S* according to:33
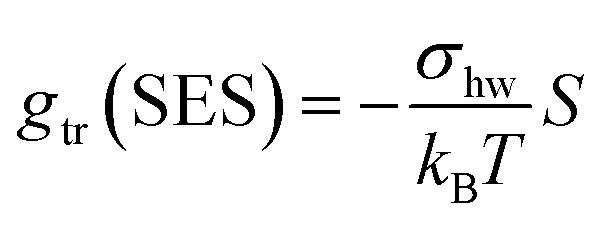
where *σ*_hw_ is again the hydrocarbon-water interfacial tension. The label “SES” is used to indicate that this way of computing *g*_tr_ differs from the traditional approach (*g*_tr_(TMT), see ref. 3 and 14) and is based on the solvent-excluded surface (SES). The latter is also known as molecular surface or Connolly surface and can be envisaged as the direct contact surface between water and hydrocarbon molecules.^50^ In contrast, the solvent-accessible surface (SAS), which is often used to model the hydrophobicity of molecules, represents the surface of closest approach of the centers of the solvent molecules to the solute surface.^51^

Indeed, there is a linear correlation of the SES values for alkanes with the transfer free energies;^52^ however, these values for the transfer free energies are different from those that are used in the traditional approach (as discussed below). Therefore, a correction factor *α* = 0.71 had to be introduced in the previous treatment.^3^ Taken together, the transfer and interfacial terms now become34
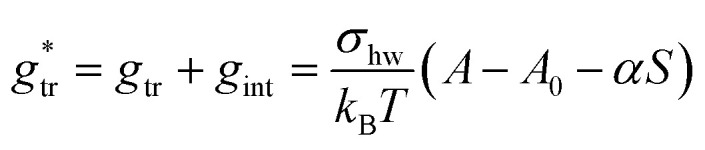
In the present work, we suggest an alternative form of 
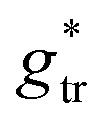
, in which the correction factor *α* is unnecessary, an effective interfacial tension *σ* is used that depends on the presence of co-solutes in the aqueous phase, and the SES of the alkyl tail is computed differently (termed S^*^, see Section 2.7):35
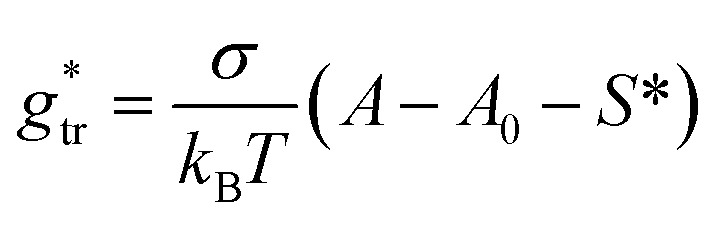


### 2.5 Structural model of micelles

As in our previous work,^3^ we do not aim here at a prediction of aggregation numbers, but rather use constraints from experiments to model the various contributions to *g*_mic_. Accordingly, we use the same geometric model^3^ that is motivated by small angle X-ray scattering (SAXS) and small angle neutron scattering (SANS) data.^29,44,45^ These data suggest^29^ that the micellar core is an oblate spheroid with minor radius *a* and major radius *b*, and the shell of detergent head groups has a thickness of 6.15 ± 0.15 Å. The experimentally determined values of *a* and *b* for DM and DDM are listed in Table 1 together with the eccentricity (or ellipticity)36
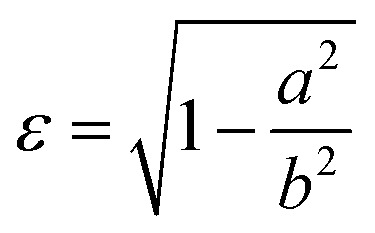
and aggregation numbers *m* derived from SAXS forward scattering intensities.^29^ Values for UDM are interpolated as described in Section 3.3. These data allow for the computation of the surface area of the micellar core per detergent molecule according to^3^37
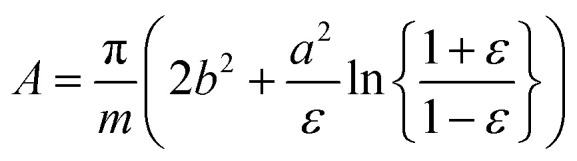


**Table 1:** Parameters used in the molecular thermodynamic modelling of *g*_mic_ according to eqn (40) and Sections 2.4 and 2.5 (*L* = 4.6 Å; *A*_0_ = 21 Å^2^; *A*_p_ = 40 Å^2^; *N* = (*n* + 1)/3.6; *R*_s_ = (*ab*^2^)^1/3^; *c*_tot_ = 54.65 M; *T* = 295 K; values of CMC(exp) from ref. 3)

*n*	10	11	12
*N*	3.0566	3.3333	3.6111
*a*/Å	12.25	13.15	14.05
*b*/Å	23.50	26.13	28.75
*ε*	0.853	0.864	0.873
*R*_s_/Å	18.91	20.78	22.65
*g*_pack_	2.05	2.27	2.49
*A*/Å^2^	57.30	56.00	50.75
S*/Å^2^	207.6	226.7	245.7
*Φ*/(N m^−1^)^−1^	−420.48	−470.55	−530.07
*g*_st_	1.20	1.25	1.55
*m*	85	106	140
*g*_mic_(exp;*m*)	−10.41	−11.67	−13.15
*g*_mic_(exp;∞)	−10.65	−11.88	−13.32
CMC(calc;*m*)/mM	1.35	0.36	0.091
CMC(calc;∞)/mM	1.44	0.37	0.087
CMC(exp)/mM	1.30 ± 0.07	0.38 ± 0.02	0.090 ± 0.005

### 2.6 Determination of total molarity

In order to connect the micellization free energy *g*_mic_ and the CMC *via*eqn (15) and (21), the total molarity *c*_tot_ of the buffer is needed. Knowing the density *ρ* of the solution as well as the molar concentrations *c* and molar masses *M* of buffer components (buffer, PIP; CaCl_2_, CaC) the total molarity can be calculated from38
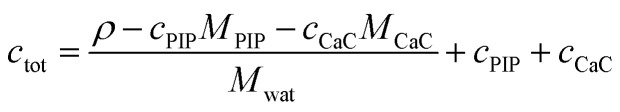
where the first term on the right-hand side is the molarity of water in the solution. For the buffer PIPES, the ratio of PIPES^−^ and PIPES^2−^ was calculated from the Henderson–Hasselbalch equation at pH = 7.0 using a p*K*_a_ of 6.76; from this ratio, the average molecular weight was determined to be *M*_PIP_ = 300.44 g mol^−1^. With the density *ρ* = 1.0135 g mL^−1^ and neglecting the small contributions from ANS and detergent, we obtain *c*_tot_ = 54.65 M.

### 2.7 Determination of solvent excluded surface area

Previously, the surface area of alkyl tails within maltoside detergents was calculated from group contributions inferred from surface areas of short alkanes (*n* ≤ 10). However, it is expected that within the detergent molecule, the alkane is partly shielded from the surrounding water by the maltose head group. Therefore, the surface of the explicit alkyl chain within a detergent molecule was determined with the following approach: three-dimensional models were built for maltose, alkanes (*n* ≤ 12) and alkyl maltosides (*n* ≤ 12) in Avogadro,^53^ and the geometry was corrected with the built-in molecular mechanics function. Based on the Cartesian coordinates, the SES areas were determined using the MSMS program^54^ with the default probe radius of 1.4 Å and the atomic radii for each atom (1.20 Å for H, 1.74 Å for C, 1.40 Å for O; according to the atmtypenumbers library). Finally, the surface area of the alkyl tail was calculated using the following linear relations (see ESI, Fig. 7†):39
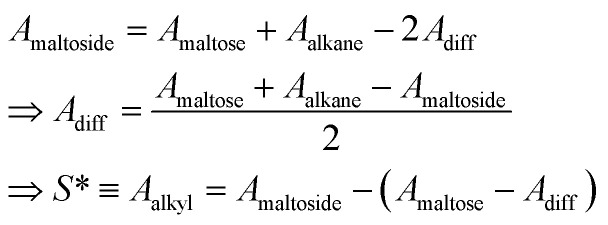


Group contributions *S*(CH_3_) and *S*(CH_3_) were obtained from a fit of S* as a function of *n* − 1, yielding *S*(CH_3_) = 36.27 Å^2^ and *S*(CH_3_) = 19.04 Å^2^.

## 3 Results

### 3.1 Definition of the critical micelle concentration

In the following, we investigate the question of whether the relationship between the CMC (that is *X*_CMC_) and the micellization free energy *g*_mic_ (in units of *k*_B_*T*) should be described by the traditional approach represented by eqn (14) (where (*X*_1_)_crit_ is the traditional “CMC”) or by our new eqn (21). We first compute *X*_CMC_ from experimental data based on eqn (15) and the graphical extrapolation procedure indicated in Fig. 1 (thereby using the *ϕ*-condition). The experimental values for the CMC, CMC(exp), have been determined previously.^3^ Then, we connect *X*_CMC_ with *g*_mic_ according to the two different definitions of the CMC, where *g*_mic_ is modelled according to Sections 2.4 and 2.5; specifically40
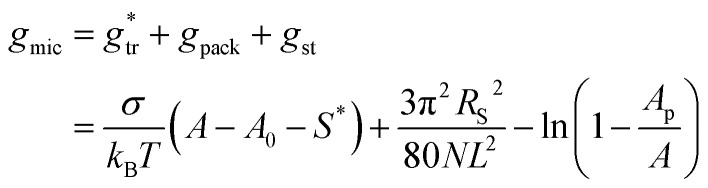


All parameters in eqn (40) can be determined from molecular thermodynamic modelling except for *σ* (see Table 1). Since *σ* depends in a complicated way on the co-solutes, it is more convenient to treat it as a fit parameter as discussed further below. To this end, we compute the quantity

**Fig. 4:**
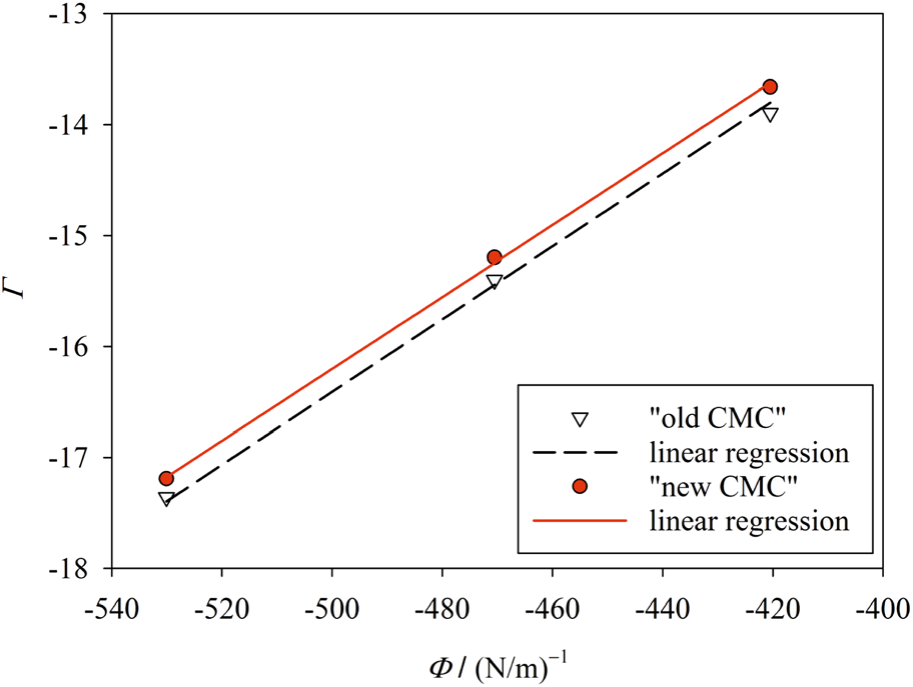
Plot of *Γ* (eqn (41)) against *Φ* (eqn (42)) for finite *m* (Table 1; “new CMC”) and for *m* → ∞ (“old CMC”). The slope of the line yields the surface tension *σ*. The fitting results are *σ* = 32.4 ± 0.055 mN m^−1^ (*R*^2^ = 0.9993) for “new CMC” and *σ* = 32.8 ± 0.095 mN m^−1^ (*R*^2^ = 0.9980) for “old CMC”. Figure made with SigmaPlot 13 (© 2014 Systat Software Inc.).

41 *Γ*: = *g*_mic_(exp;*m*) − (*g*_pack_ + *g*_st_)

where *g*_mic_(exp;*m*) is calculated from the experimentally determined CMC based on eqn (21) and (15), and plot it against the quantity42
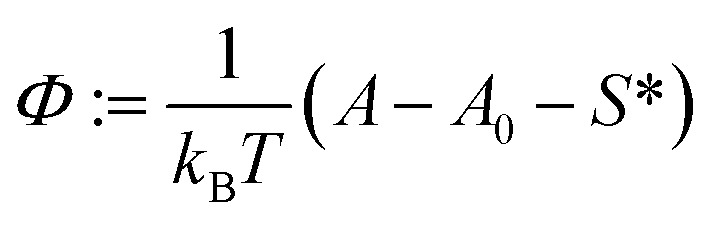
for *n* = 10, 11, 12 (Fig. 4). The slope of the plot as determined by linear regression (with zero intercept) yields *σ*. This represents the new definition of the CMC. To obtain the old definition, we consider the limit *m* → ∞, where *g*_mic_(exp;∞) = ln *X*_CMC_.

When treating *σ* as a fit parameter, it becomes a system and model dependent quantity. Accordingly, different values of *σ* are obtained when using different definitions of the CMC despite the same molecular thermodynamic model underlying the analysis (Fig. 4). The quality of the linear fit based on the new definition of the CMC is somewhat higher than that based on the old definition, but the improvement seems to be minor. However, the CMC depends exponentially on *g*_mic_. To see the improvement in predicting the CMC, we recalculate the CMC in molarity units using the fitted values of *σ* based on43
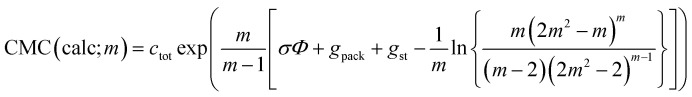
for the new definition and compare with

44 CMC(calc;∞) = *c*_tot_ exp(*σΦ* + *g*_pack_ + *g*_st_)

for the old definition (Table 1). The rms deviation between calculated and experimental CMC values is decreased from 0.08 mM for the old to 0.03 mM for the new definition. Thus, the new definition of the CMC allows for a better and more consistent modeling within the same molecular thermodynamic framework. For comparison, the rms deviation in ref. 3 was 0.16 mM for the same experimental data and the surface-based model, but without fitting *σ* (see Section 3.2).

In contrast to ref. 3, where we estimated the consequences of errors in the experimentally determined quantities *m*, *a*, and *b*, no such errors are considered in the present work (Table 1). The reason is that we found the tedious evaluation of error propagation to be of limited use. In our view, what ultimately counts is the difference between calculated and experimental CMC values, where the error margins of the latter (given in Table 1) are relevant. It can be seen that redefining the CMC (and using a correspondingly changed value for *σ*) causes the calculated CMC values for all three detergents to lie within the boundaries set by experimental uncertainties. This accuracy is achieved in none of the models based on the traditional definition of the CMC (with one exception; see below and ESI, Table 2†).

For completeness, it should be noted that the TMT approach, in which *g*_tr_ is treated separately from *g*_int_ using experimental transfer free energies, can also lead to a very good agreement between calculated and experimental CMC values with the “old” definition of the CMC. If we use *σ*_hw_ = 32.7 mN m^−1^, the re-defined geometry for UDM micelles (see Section 3.3), and eqn (14), all calculated CMC values agree within the experimental error margins with the measured values (see model 7 in ESI, Table 2†). The rms deviation even decreases to 0.01 mM. In this case, however, using eqn (21) instead of (14) does not yield better results (see model 7a).

### 3.2 Surface-based modelling of the micellization free energy

In this section we describe what led us to treat *σ* as a fit parameter. For a macroscopic interface (*e.g.* the surface of an oil droplet in water), it is known that the interfacial tension depends on the curvature of the surface.^55^ Accordingly, it has been suggested that such a dependence also exists for a microscopic interface such as the molecular surface of an alkane.^56^ The value of *σ*_hw_ = 50.0 mN m^−1^ (equivalent to ≈72 cal mol^−1^ Å^−2^) used in ref. 3 (cp. models 1 and 2 in ESI, Table 3†) corresponds to the interfacial tension of a planar macroscopic hydrocarbon-water interface. Based on their analysis of alkane transfer into water, Sharp *et al.*^56^ suggested a smaller value of 32.7 mN m^−1^ (equivalent to ≈47 cal mol^−1^ Å^−2^) to be used in conjunction with the SAS of the alkane molecules, which they ascribed to the curvature of the SAS. When we used this value for *σ*_hw_ in eqn (30) for *g*_int_ (together with the traditional modelling of *g*_tr_), we found a remarkable improvement in predicting the CMC (see model 4 in ESI, Tables 2 and 3†). Note that in this model, it is only the curvature of the surface of the micellar core that matters.

The next question was, whether the SES approach, in which *g*_tr_ and *g*_int_ are treated jointly using the solvent-excluded surface as in eqn (34), could also be improved. We first checked the influence of the revised surface areas S* (see Table 1; group contributions *S*(CH_3_) and *S*(CH_3_) listed in ESI, Table 3†) as described in Section 2.7. However, with *α* = 0.71, the rms deviation of model 3 practically doubled compared to model 2 (ESI, Table 2,†*σ*_hw_ = 50.0 mN m^−1^). One interpretation of the factor *α* is that it effectively reduced the surface tension used in *g*_tr_(SES) to a value of *ασ*_hw_ = 35.5 mN m^−1^. This could make sense, since the curvature of the surface of the alkyl tail is larger than that of the micellar core, so that *α* could be understood as a curvature correction. The problem with this interpretation, however, is that the surface of the micellar core is different from that of an oil droplet, because it is decorated with maltose head groups (see, *e.g.*, the MD simulations by Stephenson *et al.*^57^). In view of the way, such a surface interacts with water molecules, it is not clear, whether a curvature correction is reasonable in the framework of the simple model represented by eqn (30).

Another problem is that curvature corrections are discussed for the SAS rather than for the SES.^56^ Note that in our model, both the molecular surface of the alkyl tail *S* and the surface of the micellar core *S* (see eqn (37)) are to be understood as Connolly surfaces (SES). Simulations suggest that for the SES, curvature corrections are actually not necessary, at least at room temperature.^58,59^ This line of reasoning led to model 5, which employs eqn (35) with *σ* = 32.7 mN m^−1^ and *α* = 1, but does not improve the rmsd between computed and measured CMCs compared to models 2 and 4 (ESI, Tables 2 and 3†).

In another attempt to unify the description of contributions to *g*_mic_ originating from the hydrophobic effect, we assumed45
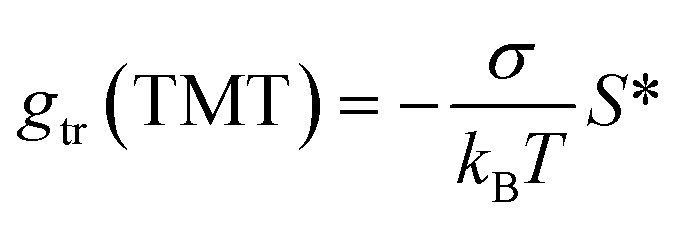
to be strictly valid, where the surface areas S* are calculated as described in Section 2.7 and *g*_tr_(TMT) are the values for *g*_tr_ used in the TMT approach. Here, *σ* becomes a fit parameter that serves to reconcile the original transfer free energies with the newly computed molecular surface areas. Using *σ* = 33.06 mN m^−1^ as obtained from this fit^60^ in model 6 leads to a further improvement compared to model 2 (ESI, Table 2†).

Although suitable values for *σ* to be used in eqn (35) are apparently very similar to the “microscopic” surface tension suggested by Sharp *et al.*,^56^ identifying *σ* with this surface tension is actually not justified. Like the transfer free energies, the value of *σ* deduced by Sharp *et al.*^56^ refers to the transfer of alkanes from pure water into a hydrocarbon phase. It is well known that co-solutes in the aqueous phase affect the transfer free energies, so that an effect of co-solutes on *σ* might be expected. Thus, we finally decided to use *σ* as a fit parameter in the procedure described in Section 3.1 to have a model at hand that is flexible enough to account for realistic buffer conditions in a biophysical context. This flexibility also allowed us to account for a refined definition of the CMC.

### 3.3 Improved micelle geometry for undecyl maltoside

In our earlier work,^3^ we used *m* = 110 as the aggregation number of UDM micelles. This value roughly corresponds to a linear relationship between *m* and the alkyl chain length *n* in accordance with claims in the literature.^40^ However, when values for *m* from several sources are considered,^29,40–43^ they together suggest an exponential dependence of *m* on *n* (see ESI, Fig. 8, Table 4†):

46 *m* = 5.4276 e^0.2699*n*^

For DM, UDM, and DDM, this equation predicts aggregation numbers of 80.5, 105.7 and 137.7, respectively, in good agreement with the experimental values found by Lipfert *et al.*^29^ for DM and DDM that we already used for the molecular thermodynamic modelling. Based on these findings, we adopted the value *m* = 106 in our refined modelling of UDM (*cf.*Table 1).

Experimental SAXS data provide values for *a* and *b* of oblate spheroidal micelles of octyl maltoside (OM), DM, and DDM.^40^ The lengths of both axes seem to be correlated to the alkyl chain length in an almost perfectly linear way (see ESI, Fig. 9†), which allows determining these parameters for UDM by linear interpolation as listed in Table 1.

### 3.4 Application to other alkyl maltosides

**Fig. 5:**
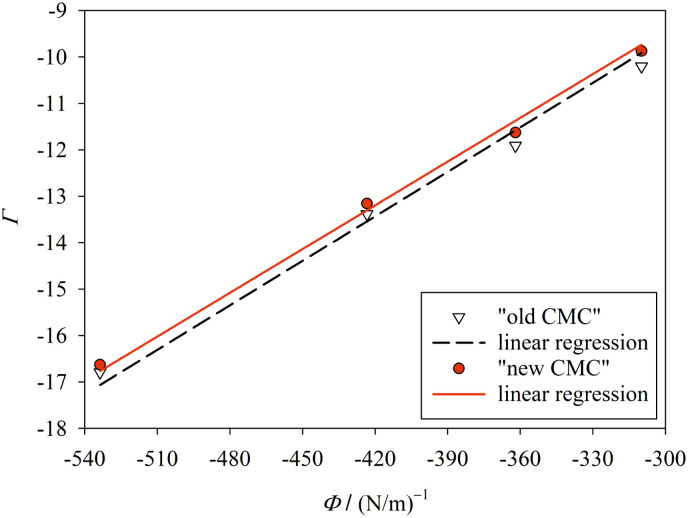
Same as in Fig. 4, but for the experimental CMC data of Alpes *et al.*^32^ The fitting results are *σ* = 31.4 ± 0.2 mN m^−1^ (*R*^2^ = 0.9950) for “new CMC” and *σ* = 32.0 ± 0.4 mN m^−1^ (*R*^2^ = 0.9873) for “old CMC”. Figure made with SigmaPlot 13 (© 2014 Systat Software Inc.).

In order to further test our method, we searched the literature for published values of the CMC of alkyl maltosides.^32–39^ However, since we require the CMC to be measured by a fluorescence technique, which is compatible with the *y*-condition, only the data by Alpes *et al.*^32^ (with one data point taken over from De Grip and Bovee-Geurts^34^) could be analyzed. Alpes *et al.*^32^ determined the CMC of OM, nonyl maltoside (NM), and DM in 150 mM KCl by using the dye 1,6-diphenyl-1,3,5-hexatriene (while De Grip and Bovee-Geurts^34^ used ANS for DDM under similar conditions). These data can be described well by our model (Fig. 5, ESI, Table 5†), with the new definition of the CMC performing slightly better than the old definition. The values for *σ* are smaller than those from our data (cp. Fig. 4), which can be traced back to the differences in solution conditions, and the value for the new definition of the CMC is consistently smaller than that for the old definition.

**Fig. 6:**
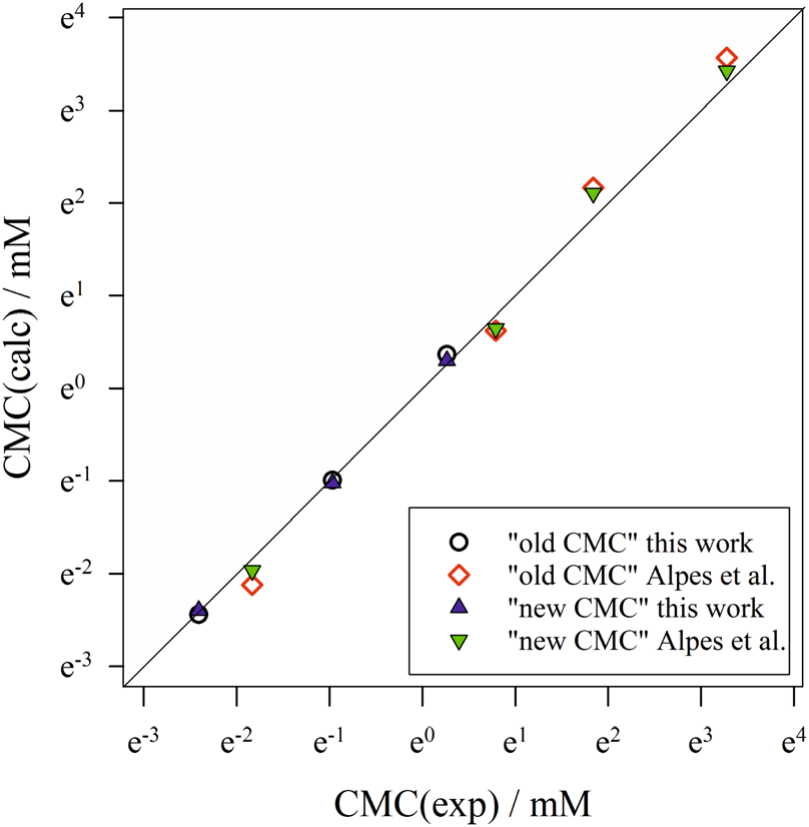
Correlation of calculated CMC values according to the “old” and “new” definitions of the CMC with experimental CMC values from both data sets (this work and Alpes *et al.*^32^). The symbol size represents the estimated experimental error, while the straight diagonal line represents perfect correlation. Figure made with SigmaPlot 13 (© 2014 Systat Software Inc.).

To summarize, we show in Fig. 6 the correlation between computed CMC values according to both definitions of the CMC and the experimental CMC values from both data sets in a log–log plot. The molecular thermodynamic model based on eqn (40) yields a very good description of the CMC of alkyl maltosides. For almost all data points, the new definition of the CMC results in a better description of the experiments than the old definition as can also be seen from the relative deviations [CMC(calc) − CMC(exp)]/CMC(exp) (see ESI, Fig. 10†). Although the refined definition of the CMC results only in relatively slight improvements, we consider it relevant to employ a definition of the CMC that is consistent with the experimental procedure in order to reliably evaluate the molecular thermodynamic model.

## 4 Discussion

### 4.1 A new angle on the critical micelle concentration

What we here call the “new” definition of the CMC to have a handy notion, in fact, dates back to the 1955 paper by Phillips,^20^ who introduced the idea of taking the third derivative of a quantity as a function of the total detergent concentration to define the CMC. So what is actually new in our treatment?

First of all, Philips’ idea appears to have only a relatively minor impact on detergent research as the overwhelming majority of works in the field relies on the “old” definition in the spirit of eqn (14) as nicely explained by Israelachvili^11^ (see also the seminal work by Nagarajan and Ruckenstein^14^). A rare example of a direct implementation of Phillips’ approach is the work by Garcia-Mateos *et al.*,^61^ who investigated conductivity data of ionic detergents. In the sequel, the method was further developed mostly for this kind of data.^62–64^

Secondly, in Phillips’ approach, it is rigorously assumed that the measured quantity *ϕ* is “ideal” in the sense that the breaking point in the *ϕ*(*x*) curve directly reflects the molecular behaviour. This assumption can be made more precise by writing for the measured observable47
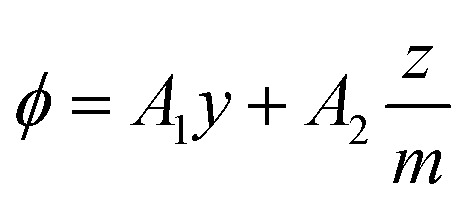
where *A*_1_ and *A*_2_ are proportionality constants, while *m*, *y*, and *z* are defined as above. It is noteworthy that applying the *ϕ*-condition in eqn (16) directly to *ϕ* as defined by eqn (47) leads to the ratio of monomer to total detergent concentration at the breaking point in eqn (20) (see ESI Text S2†). It has been noticed, however, that the measurable quantity *ϕ* not necessarily obeys eqn (47) and it would be more consistent to apply the concept of the third derivative directly to the monomer concentration *y*. This idea was pursued by Al-Soufi *et al.*,^31^ who developed empirical methods to reconcile CMC data obtained from different measurement techniques. In contrast, we employed the *y*-condition in a purely theoretical framework. By virtue of implicit differentiation, we could derive eqn (20) without any reference to a measurable quantity and exploit the *y*-condition to link the CMC to the micellization free energy in a rather general way (see eqn (21)). Only in a second independent step, we showed that in the particular case that *ϕ* is the fluorescence intensity of a suitable dye, the breaking points of the experimental and the theoretical curves coincide. We note that for other experimental methods to determine the CMC, this coincidence remains to be investigated. Such an investigation may help to reconcile CMC values obtained from various methods, which often yield different results or employ rather loose definitions of the CMC as discussed by Al-Soufi *et al.*^31^

Thirdly, Phillips applied his method to ionic detergents bearing an effective charge and interacting with counter ions.^20^ Interestingly, he arrived at a different formula for the ratio of monomer to total detergent concentration at the breaking point (see his eqn (8b)) and for the relationship between micellization free energy and CMC (see his eqn (12)). We have to conclude that our results are not general, but only apply to nonionic or zwitterionic detergents, where no counter ions have to be taken into account. Since under the constraint that eqn (47) holds, our method is equivalent to that of Phillips, it might be possible to arrive at his equations without recourse to a specific “ideal” experimental quantity. This possibility remains to be investigated.

Fourthly, it is noteworthy that many works even when employing Phillips’ idea, assume that the micelle concentration is practically zero below and even at the CMC. By combining eqn (15) and (20) with the mass balance of the detergent, we obtain for the micelle concentration [micelle] at the CMC:48
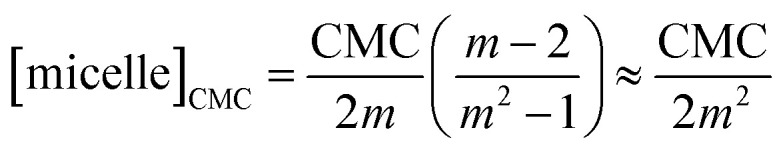


It is easy to see from this equation that [micelle]_CMC_ is at least four orders of magnitude smaller than the CMC and, indeed, goes to zero for *m* → ∞. Thus, neglecting the micelle concentration at (and below) the CMC seems justified. However, the concentration of detergent bound to micelles scales with CMC/2*m* and thus is only two orders of magnitude smaller than the CMC. It should be stressed that the definition of the CMC to be employed is actually not a matter of taste: if the fluorescence technique is used, the detergent concentration at the breaking point of the titration curve is taken as the CMC. Then, according to our analysis, depending on the aggregation number *m*, 0.3 to 1.0% of detergent is bound to micelles at the CMC. For example, for *n* = 8, this amounts to about 0.3 mM, whereas for *n* = 12, it is only 0.3–0.6 μM depending on the solution conditions.

Fifthly, it is also usually assumed that the monomer concentration equals the CMC above the CMC. This, too, is an approximation: If 
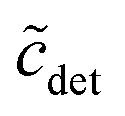
 is the total detergent concentration divided by the CMC and 
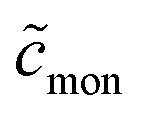
 the monomer concentration divided by the CMC, it follows from eqn (22) that49
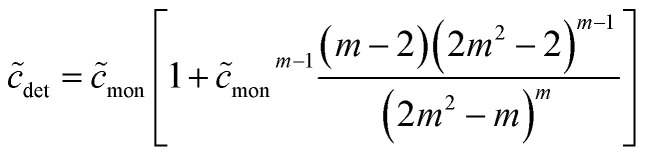


From eqn (49), we can estimate 
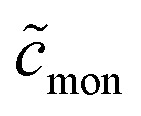
 equals 1.143, 1.084, and 1.054, respectively, for OM, DM, and DDM, if 
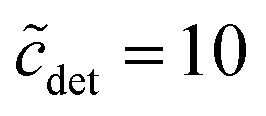
. Thus, depending on *n* (and hence *m*), the monomer concentration is between 5 and 14% above the CMC, if the total detergent concentration is ten times the CMC.

### 4.2 Simplifying assumptions

Despite the good performance of our model, it should be kept in mind that it relies on a number of approximations, which may require a critical analysis in future refinements. We shall briefly discuss only two of these approximations. One is the use of the ideal solution model for the entropy of mixing. This model ignores the differences in the sizes of the various species present in the solution. Nagarajan^26^ investigated the performance of various entropy models in predicting the CMC and other detergent properties in a molecular thermodynamic framework. We note that in this particular work, Nagarajan practically uses the *y*-condition by defining the CMC as “the total surfactant concentration at which a sharp transition is observed in a plot of the total surfactant concentration *versus* the singly dispersed surfactant concentration”,^26^ corresponding to our *x*(*y*) curves (*cf.*Fig. 3A). The main conclusion from Nagarajan's analysis is that the ideal solution model, while failing in the prediction of the solution phase behavior, provides a good prediction of the CMC.

Size differences between molecular species in the solution are also central to the analysis of transfer free energies by Sharp *et al.*^56^ that led to a revision of the “microscopic” surface tension. It can be assumed that molecular volume effects are as important for the micellization free energy as they are for partition coefficients. It is then somewhat surprising that the neglect of these volume effects in our treatment yields effective surface tensions that are similar to the value inferred by Sharp *et al.*^56^ We will come back to this problem in Section 4.3.

The second important approximation is the assumption of a fixed aggregation number *m*, which corresponds to using a mass action model of micelle formation (*cf.* ESI Text S2, eqn (S5)†). We note that this assumption was not made by Nagarajan,^26^ but is inherent to all the methods discussed above in Section 4.1. In general, the micellar size distribution (see eqn (11)) can have a complex dependence on the total detergent concentration, which is determined by the standard chemical potential difference *μ*^0^_*ν*_ − *μ*^0^_1_. In this respect, important cornerstones regarding DDM are provided by the seminal work of Warr *et al.*,^30^ who demonstrated some peculiar properties of sugar surfactants. Aqueous disaccharide surfactant systems show a strikingly simple phase diagram with an isotropic micellar solution extending to very high total surfactant concentrations (>40 wt% in the case of DDM). The micelle size distribution is relatively insensitive to temperature. It is concluded from viscosity measurements that there is no change in micelle size with increasing concentration. However, the picture emerging from fluorescence quenching experiments to determine the aggregation number appears to be somewhat more complicated. Warr *et al.*^30^ determined the (mean) aggregation number of DDM to be 111 ± 10 for 
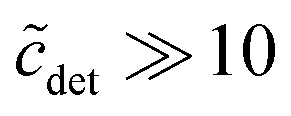
. In the region 
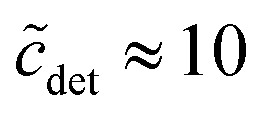
, it is only between 90 and 100, and at smaller detergent concentrations, it even drops below 80. At the same time, the aggregation number distribution shows a fairly concentration-independent rms deviation of about 40 and practically no skewness. The value of the rms deviation is considered too high for the micelles to be spherical. These data are in reasonable agreement with those of Lipfert *et al.*,^29^ if one takes into account that in the SAXS experiments, the aggregation number is somewhat higher (140 ± 10) and its determination is obscured by intermicellar repulsion at higher concentrations. The lowest concentration investigated by Lipfert *et al.*^29^ is in fact 
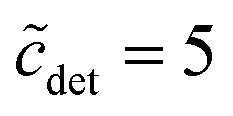
 (for DDM), and aggregation numbers are extrapolated to zero concentration (which are the values that we are using in our modeling). Since detergent concentrations applied in membrane protein research are typically below ten times the CMC, the question of a possible dependence of the sizes of micelles (and eventually the sizes of detergent belts in PDCs^8–10^) in this region is of interest (*cf.* the discussion in ref. 3). Unfortunately, this concentration range is hardly resolved in experiments that determine the aggregation number. At least, the data by Warr *et al.*^30^ point to the possibility that the aggregation numbers of alkyl maltosides, while being fairly constant at higher concentrations, may be somewhat smaller in the vicinity of the CMC. This problem will have to be taken into account in future refinements of the model. Meanwhile, the mass action model, although not able to resolve the size variation issue, may still serve as a valuable tool to study the influence of various factors on the CMC.

### 4.3 Understanding the microscopic surface tension

How to interpret the parameter *σ* in eqn (40)? It clearly has the dimension of a surface tension as it originates from a surface-based description of the free energy change due to the transfer of hydrophobic parts of the detergent molecule from water into the micelle. However, when determined by a fit as shown in Fig. 4, it is also affected by limitations of the molecular thermodynamic model that are not related to molecular surfaces. The quantity *Γ* not only contains the experimental error of the CMC, but also deficiencies of modeling *g*_pack_ and *g*_st_ (*cf.*eqn (41)). Furthermore, it depends on the way the CMC is defined. Thus, a physical interpretation of *σ* seems difficult.

If we interpret *σ* as a surface tension, we may ask what the meaning is of a surface tension at the molecular scale. In our study, we made two interesting observations: (i) the values of *σ* obtained by the fit are very close to the “microscopic” surface tension determined by Sharp *et al.*^56^ (ii) the CMC is very sensitive to the value of *σ*. The latter point can best be seen from a comparison of the cases *n* = 10 (DM) and *n* = 12 (DDM) in our data set and that of Alpes *et al.*^32^ (see the data points in the lower left corner and in the center of Fig. 6). For example, Alpes *et al.*^32^ (or actually De Grip and Bovee-Geurts^34^) obtain a CMC of 0.16 mM for DDM, whereas we obtain 0.09 mM under our buffer conditions.^3^ Both values can be reconciled by changing only *σ*. Although there are slight differences in the temperature and the total molarity, all other parameters entering *g*_mic_ except for *σ* are the same including those entering *g*_pack_ and *g*_st_ as well as *A*, *A*_0_, and S* (*cf.*Table 1 and ESI Table 5†).

Why is *σ* similar to the microscopic surface tension determined by Sharp *et al.*?^56^ Note that we employ the SES rather than the SAS used by Sharp *et al.* Based on the surface increment of a methylene group, which is 19.04 Å^2^ in our SES calculation (see Section 2.7 and ESI Table 3†) and ≈ 29 Å^2^ for the SAS,^56^ one can estimate that the SAS is larger than the SES by a factor of approximately 1.5. Thus, if we had used the SAS, we would have obtained a value of *σ* = (32.4/1.5) mN m^−1^ = 21.6 mN m^−1^ from the fit. It follows that our value of *σ* is actually too small.

A possible reason for confusion is that the increase of the surface tension due to considering molecular volume effects in the analysis of transfer free energies by Sharp *et al.*^56^ is by a factor of 32.5/21.5 ≈ 1.5, which accidently is the same as the SAS/SES ratio. (It may add to the confusion that the conversion factor from cal mol^−1^ Å^−2^ to mN m^−1^ is also ≈ 1.5.) Thus, using the SES instead of the SAS compensates for neglecting molecular volume effects in the entropy of mixing. Indeed, if the transfer free energies of alkanes are corrected for volume effects and correlated with the SES, a value of *σ* = 47.9 mN m^−1^ is obtained.^52^ It is remarkable that the latter value is very close to the surface tension of a macroscopic alkane–water interface.^3^

It follows that the ideal solution model for the entropy of mixing as represented by eqn (7) could be the cause of a systematic error that results in values of the effective surface tension *σ* in eqn (40) that are too low by a factor of about 2/3. To further investigate this problem and to learn more about the effects of co-solutes on *σ*, we drafted a non-ideal solution model (see ESI Text S3†). In this model, molecular volume effects are taken into account following the work of Hildebrand,^65^ while the interaction of detergent with one type of co-solute is described in a mean field approach akin to the Bragg-Williams approximation.^66^ The equilibrium constant of the mass action model then becomes50
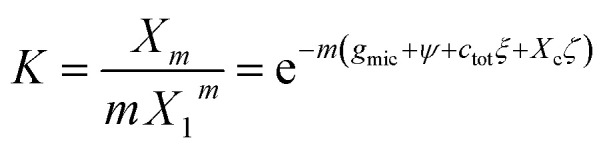
where additional terms appear in the exponent due to non-ideal behavior. The quantity *ψ* originates from a term 1 − *m* as well as a necessary correction to express *K* in terms of volume fractions rather than mole fractions and is practically the same for all detergents (*i.e. ψ* ≈ −4.2, see ESI, Table 6†). *mξ* represents the difference in molar volume between *m* detergent monomers and a micelle with aggregation number *m*, whereas *mζ* is the difference in interaction with the co-solute between *m* detergent monomers and a micelle (*X*_c_ being the mole fraction of the co-solute). *ξ* and *ζ* are difficult to quantify, but are likely small.

To take non-ideality into account in the analysis of experimental CMCs, we can define *Γ* and *Φ* as in eqn (41) and (42), respectively, and compute *g*_mic_(exp;*m*) from experimental data on the basis of eqn (21) and (15). Then, we have to find *σ* by fitting

51 *Γ* − *ψ* − *c*_tot_*ξ* − *X*_c_*ζ* = *σΦ*

Since *ψ* is practically constant, it cannot affect the slope of the plot. Any change of *σ* has to originate from *ξ* or *ζ*. Thus, eqn (51) shows, why *σ* is expected to depend on the co-solutes: if *ζ* for a particular solute is different for the various detergents, it will affect the slope of the plot. Likewise, any dependence of the volume difference between *m* detergent monomers and a micelle on the alkyl chain length *n* will affect the fitting results. However, it remains an open question of whether these effects are large enough to change *σ* by a factor of the order of 2/3. So, the relation between *σ* and the surface tension of a macroscopic alkane–water interface remains unclear. On the other hand, the slightly different values of *σ* obtained from the two different sets of experimental data could well originate from co-solute effects due to *ζ*.

Further quantification of *ξ* and *ζ* is very challenging and clearly beyond the scope of the present paper. Therefore, in the absence of a reliable model for the non-ideality of the solution, we can specify co-solute effects in our model with a fitted, solution-dependent *σ* in a kind of semi-empirical way. Is it possible to describe the effects of all types of co-solutes in this way? The answer is decisively no. Our model rests on experimental information about the size and geometry of the micelles. In implementing this information, we make the tacit assumption that the co-solutes do not alter these micellar properties. However, this assumption is not always tenable. For example, small amphiphilic molecules like heptane-1,2,3-triol are known to decrease micelles^67,68^ and are employed in membrane protein crystallization with the goal to downsize the detergent belt in the PDC.^69,70^ Similar effects can be expected for glycerol.^71,72^ In such cases, where a co-solute tends to enter the micelles to an appreciable extent, the model for *g*_mic_ would have to be modified to directly contain a term depending on the concentration of the co-solute. However, even macromolecular co-solutes, which do not enter the micelles, can attach to either detergent monomers or micelles. In those cases, it would likewise be necessary to modify the equation for *g*_mic_. Problems of this type will be discussed in a forthcoming publication dealing with PEG.

### 4.4 Modelling the packing free energy

The analytical expression for the packing free energy in eqn (31) was introduced by Nagarajan and Ruckenstein^14^ based on the theoretical work by Semenov, who formulated an analytical expression for the deformation free energy of block-copolymers within spherical microdomains.^73^ Nagarajan and Ruckenstein defined the alkyl chain to be composed of *N* segments with the length *L* = 4.6 Å, following the lattice definition used by Dill and Flory in their description of the micellar core.^74^ The number of segments *N* is defined as *N* = (*n* + 1)/3.6, where 3.6 is the number of methylene groups per lattice site. *R*_s_ in eqn (31) is the radius of the aggregate assumed to be spherical. For the present modelling of ellipsoidal micelles, *R*_s_ was defined as the radius of a sphere with the same volume as the micellar core:^3^*R*_s_ = (*ab*^2^)^1/3^.

Interestingly, despite the non-spherical micelle shape, using eqn (31) results in a model that predicts the CMC very well. In order to understand why this might be the case, it is interesting to examine a proposed correction for ellipsoidal micelles. Iyer and Blankschtein used a statistical-mechanical model to compute the packing free energy for prolate and oblate ellipsoidal geometries.^25^ In contrast to spheres, cylinders, and bilayers, the computationally expensive determination of the packing free energy for ellipsoids cannot be condensed into an analytical expression. Therefore, a direct application of their method to the micelles of alkyl maltosides was not feasible within the scope of the present paper.

However, the results from Iyer and Blankschtein^25^ indicate that the micelle shape has a significant impact on *g*_pack_ only for small micelles. For large oblate spheroidal micelles with a minor axis *a* = 0.95*l*_c_ (where *l*_c_ is the maximum extension of the alkyl chain), the value of *g*_pack_ only changed by less than 5% for a ratio *b*/*a* = 2 compared to a sphere (*b*/*a* = 1), which was attributed to the lower curvature of large micelles and the correspondingly lower conformational constraints. For the alkyl maltosides studied in the present work, *b*/*a* ≈ 2 and *a* ≈ 0.88*l*_c_, which is similar to the above values. Moreover, the micelles of alkyl maltosides are even larger than those investigated by Iyer and Blankschtein, which could imply that the movement of alkyl chains is even less constrained and correspondingly, the effect of curvature on the value of *g*_pack_ becomes negligible. This, in turn would explain why eqn (31) allows predicting the CMC well, although it strictly applies only to spheres.

## 5 Conclusions and outlook

A precise definition of the CMC is possible by setting to zero the third derivative of the concentration of detergent monomers as a function of the total detergent concentration. When combined with a mass action model for micelle formation (without counter ions), this definition results in controllable analytic formulae for the concentration ratio of monomers to total detergent at the CMC and the relationship between the CMC and the free energy of micellization. These equations differ from those obtained earlier by Phillips^20^ for ionic detergents and do not require the assumption of an ideal measurable quantity obeying eqn (47). The fluorescence enhancement of ANS (and similar probe dyes) is a suitable observable, for which the breaking point in the experimental titration curve coincides with the breaking point in the theoretical curve, thus allowing for a direct determination of the CMC according to the above definition.

When applied to a series of *n*-alkyl-β-d-maltosides C_*n*_G_2_ with alkyl chain lengths *n* ranging from 8 to 12, the more precise definition of the CMC allows for demonstrating the good performance of a molecular thermodynamic model, in which the free energy of micellization is given by eqn (40). In this model, *σ* is a fit parameter with the dimension of a surface tension, which represents those parts of the micellization free energy that are due to a change in the area of hydrophobic molecular surfaces in contact with the aqueous phase. All other parameters of the model are inferred from a consideration of the micelle geometries based on independent experimental data and molecular structure. It turns out that different experimental conditions due to co-solutes that do neither attach to detergent monomers nor to micelles to a significant extent can be accounted for by adapting only *σ*. However, the relation of *σ* to macroscopic surface tension concepts remains unclear.

The present work sets the stage for future applications of the theoretical approach in biophysics and biochemistry, but also in the more general field of detergency, where it is still an unsolved problem to reconcile CMC data from different experimental methods. Our contribution provides a first step in this direction by demonstrating the way to link theory and experiment for the case of one particular experimental method. Applications to other methods and other types of detergents (*e.g.*, surface tension measurements and gemini surfactants^75^) will follow. The model will also be applied to problems in the context of membrane protein research such as the influence of PEG on the CMC or the formation of PDCs.

## Conflicts of interest

There are no conflicts to declare.

## Supplementary Material

RA-013-D2RA07440K-s001
