# Supplementary material for: Refined definition of the critical micelle concentration and application to alkyl maltosides used in membrane protein research
Source: RSC Adv. 2023 Mar 22;13(14):9387–401. doi: 10.1039/d2ra07440k (PMC10031436; doi:10.1039/d2ra07440k)
Supplement: RA-013-D2RA07440K-s001 [file RA-013-D2RA07440K-s001.pdf]

Electronic Supplementary Material (ESI) for RSC Advances.
This journal is © The Royal Society of Chemistry 2023

SUPPLEMENTARY INFORMATION

Refined definition of the critical micelle concentration and application to alkyl maltosides used in membrane protein research

Adrian Bothe,[a] Athina Zouni [a] and Frank Müh*[b]

[a] *Institut für Biologie, Humboldt Universität zu Berlin, Leonor-Michaelis-Haus, Philippstrasse 13, D-10095 Berlin, Germany.*

[b] *Institut für Theoretische Physik, Johannes Kepler Universität Linz, Altenberger Strasse 69, A-4040 Linz, Austria*

* *E-mail: frank.mueh@jku.at*

ESI Tables

Table 2 Comparison of experimental CMC values (mM) for DM, UDM, and DDM with calculated values based on different models (see main text and Table 3) using $c_{\mathrm{tot}} = 54.65\ \mathrm{M}$ and the traditional definition of the CMC, in which $g_{\mathrm{mic}} = \ln X_{\mathrm{CMC}}$ (except for model 7a). Data for model 9 are the same as in Table 1.

Model [a]	DM	UDM	DDM	rmsd [b]
exp.	1.30 ± 0.07	0.38 ± 0.02	0.090 ± 0.005	–
1	6.10	1.12	0.304	2.81
2	1.12	0.18	0.037	0.16
3	1.86	0.28	0.069	0.33
4	1.31	0.30	0.094	0.05
5	1.71	0.37	0.110	0.24
6	1.46	0.31	0.090	0.10
7	1.31	0.37	0.094	0.01
7a [c]	1.03	0.30	0.080	0.16
8	1.46	0.38	0.090	0.09
9	1.44	0.37	0.087	0.08

[a] (1) TMT model with g_{tr} from group contributions based on transfer free energies and g_{int} computed using the macroscopic interfacial tension σ_{hw};[1] (2) surface-based approach (SES) as in ref. 1 using the macroscopic interfacial tension σ_{hw} in g^{*}_{tr}; (3) SES* approach, i. e., SES approach using the refined molecule surfaces S^{*} (see main text); (4) TMT approach as in 1, but using the microscopic interfacial tension σ;[2] (5) SES* approach with the same value for the interfacial tension as in 4; (6): SES* approach with $\sigma = 33.06\ \mathrm{mM\ m^{-1}}$;[3] (7) model 4 with re-defined micelle geometry for UDM (see main text); (8) model 6 with re-defined micelle geometry for UDM (see main text); (9): New model with fitted interfacial tension σ (Fig. 4, "old CMC") and re-defined micelle geometry for UDM (see main text).

[b] Root mean square deviation in mM.

[c] Model 7a has the same g_{mic} as model 7, but employs eq. (21) instead of eq. (14).

Table 3 Parameters used in the various models listed in Table 2.

Model [a]	1	2	3	4	5	6	7	8	9
σ_{hw} / mN m^{-1}	50.0 ± 1.0	50.0 ± 1.0	50.0 ± 1.0	32.7 [b]	32.7 [b]	33.06 ± 0.35	32.7 [b]	33.06 ± 0.35	–
σ / mN m^{-1}	–	–	–	–	–	–	–	–	32.80 ± 0.095 [c]
$g_{\mathrm{tr}}(\mathrm{CH_3})$ [d]	– 3.72	–	–	– 3.72	–	–	– 3.72	–	–
$g_{\mathrm{tr}}(\mathrm{CH_2})$ [e]	– 1.45	–	–	– 1.45	–	–	– 1.45	–	–
α [f]	–	0.71	0.71	–	1	1	–	1	1
$S(\mathrm{CH_3})$ / Å^2 [g]	–	35.02	36.27	–	36.27	36.27	–	36.27	36.27
$S(\mathrm{CH_2})$ / Å^2 [h]	–	19.85	19.04	–	19.04	19.04	–	19.04	19.04

[a] See footnote a to Table 2. Models 7, 8, and 9 employ a refined geometry for UDM.
[b] Value from Sharp et al. (ref. 2); no error given.
[c] From fit (see Fig. 4, "old CMC").
[d] Group contribution from methyl group to the transfer free energy in units of $k_{\mathrm{B}}T$ (cp. ref. 1; error ± 0.07).
[e] Group contribution from methylene group to the transfer free energy in units of $k_{\mathrm{B}}T$ (cp. ref. 1; error ± 0.02).
[f] Correction factor; see eq. (34). $\alpha = 1$ means that no correction factor is used in g_{tr}^{*}; see eq. (35).
[g] Group contribution from methyl group to the molecular surface. Values in model 2 are from ref. 1, the other values are from the present work.
[h] Group contribution from methylene group to the molecular surface. Values in model 2 are from ref. 1, the other values are from the present work.

Table 4 Average values for the aggregation number m of alkyl maltoside micelles for different lengths n of the alkyl chain, compiled from several sources.

n	m	Reference
8	47.7 ± 5.7	Oliver et al., 2013;[4] Kunji et al., 2008.[5]
9	60.5 ± 5.5	Kunji et al., 2008.[5]
10	82.1 ± 9.1	Lipfert et al., 2007;[6] Oliver et al., 2013;[4] Kunji et al., 2008.[5]
11	105	Kunji et al., 2008.[5]
12	134.4 ± 11.6	Lipfert et al., 2007;[6] Oliver et al., 2013;[4] Kunji et al., 2008;[5] Tummino and Gafni, 1993;[7] Jumpertz et al., 2011.[8]
13	186	Kunji et al., 2008.[5]

Table 5: Parameters used in the molecular thermodynamic modelling of g_{mic} according to eq. (40) and Sections 2.4 and 2.5 ($L = 4.6$ Å; $A_0 = 21$ Å^2; $A_{\mathrm{p}} = 40$ Å^2; $N = (n+1)/3.6$; $R_{\mathrm{s}} = (ab^2)^{1/3}$; $c_{\mathrm{tot}} = 55.32$ M corresponding to 150 mM KCl;[a] $T = 293$ K). Values of $\mathrm{CMC(exp)}$ from Alpes et al.[9] with the value for DDM ($n = 12$) taken over from De Grip and Bovee-Geurts.[10] Experimental error assumed to be 5.6 %.

n	8	9	10	12
N	2.5000	2.7778	3.0566	3.6111
a / Å	10.45	11.35	12.25	14.05
b / Å	18.25	20.88	23.50	28.75
ε	0.820	0.839	0.853	0.873
R_{s} / Å	15.16	17.04	18.91	22.65
g_{pack}	1.61	1.83	2.05	2.49
A / Å^2	65.12	63.13	57.30	50.75
S^* / Å^2	169.5	188.6	207.6	245.7
Φ / (N/m)$^{-1}$	–309.86	–361.98	–423.35	–533.69
g_{st}	0.95	1.00	1.20	1.55
m	47	62	85	140
$g_{\mathrm{mic}}(\mathrm{exp}; m)$	–7.31	–8.80	–9.91	–12.59
$g_{\mathrm{mic}}(\mathrm{exp}; \infty)$	–7.64	–9.08	–10.13	–12.75
$\mathrm{CMC(calc}; m)$ /mM	30.65	8.21	1.91	0.14
$\mathrm{CMC(calc}; \infty)$ /mM	35.35	8.74	1.87	0.12
$\mathrm{CMC(exp)}$ / mM	26.50 ± 1.48	6.30 ± 0.35	2.20 ± 0.12	0.16 ± 0.01

[a] Computed from $c_{\mathrm{tot}} = (\rho - c_{\mathrm{KCl}} M_{\mathrm{KCl}})/M_{\mathrm{wat}} + c_{\mathrm{KCl}}$ with $\rho = 1.005$ g/mL, $c_{\mathrm{KCl}} = 0.15$ M, $M_{\mathrm{KCl}} = 74.551$ g/mol, and $M_{\mathrm{wat}} = 18.015$ g/mol.

ESI Figures

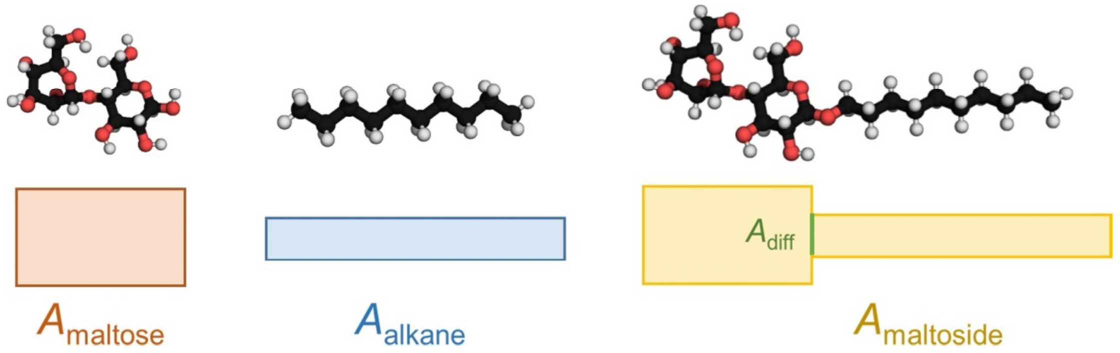

Fig. 7 Determination of the surface area of alkyl tails from alkyl maltosides. The solvent-excluded surface of molecular models of maltose, alkanes and alkyl maltosides was measured using the MSMS script.[11] Then, using the linear relations in eq. (39) of the main text, the surface of the alkyl tail was calculated by subtracting the maltose portion of the surface from the surface area of the whole detergent molecule. The molecular models in this figure do not represent the solvent-excluded surface. Instead, a ball and stick model is used with coloring according to the CPK convention.

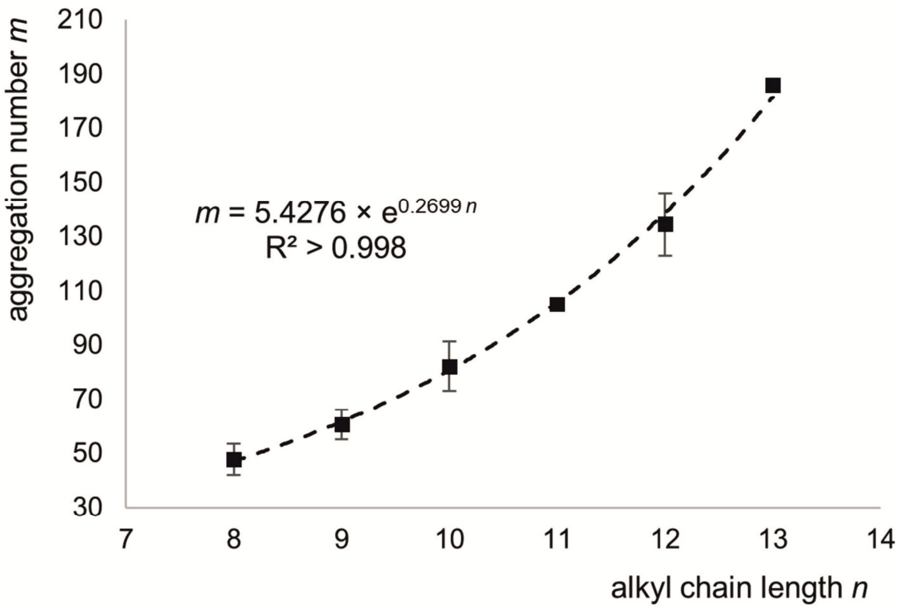

Fig. 8 When experimental values for the aggregation number m of alkyl maltosides are plotted against the alkyl chain length n, an exponential dependence is obtained. The data was compiled from several sources (see Table 4).

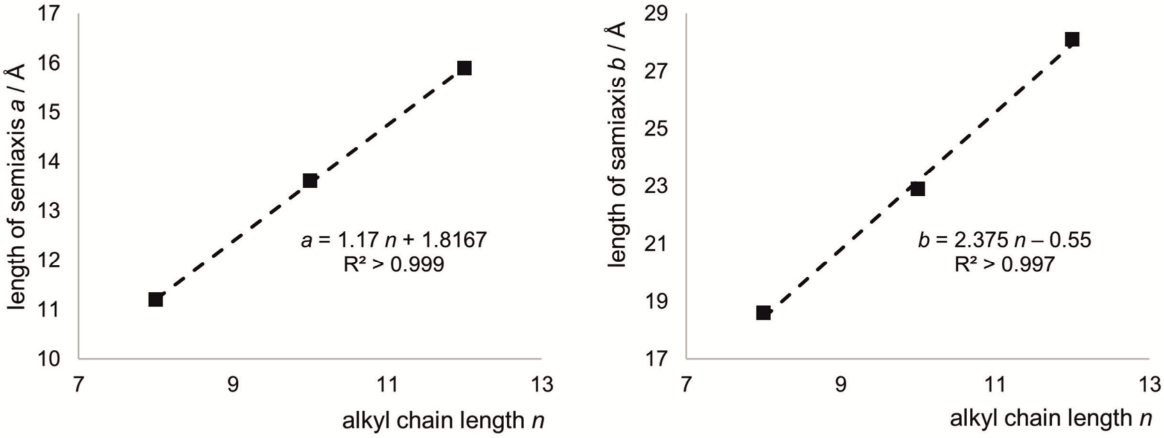

Fig. 9 Lengths of the semiaxes a and b of an ellipsoid describing the micelles of octyl-, decyl-, and dodecyl maltoside based on SAXS.[4] Due to the nearly perfect linear relationship between the length of each semiaxis and the alkyl chain length n, the values for undecyl maltoside ($n = 11$) can be calculated by linear interpolation.

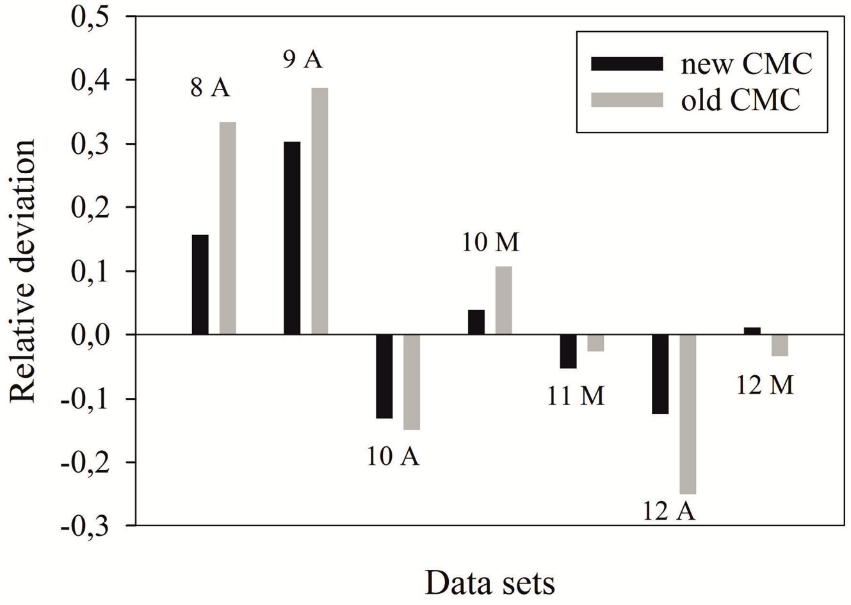

Fig. 10 Relative deviation $[\mathrm{CMC(calc)} - \mathrm{CMC(exp)}]/\mathrm{CMC(exp)}$ for the different data sets and both definitions of the CMC. The numbers refer to the alkyl chain length n and the letters to the source of experimental data (A: Alpes et al., 1986;[9] M: Müh et al. 2015[1]).

ESI Text

S1: Modelling the steric free energy g_{st}

The steric free energy accounts for the repulsion between the polar head groups of adjacent detergent molecules within the micelle and is generally considered to be the component that limits the micelle growth to a certain aggregation number. The mathematical expression for g_{st} in eq. (32) of the main text was suggested based on the van-der-Waals equation of state:[12, 13]

$$P = \frac{RT}{v-b} - \frac{a}{v^2} \tag{S1}$$

where P is the pressure, R the gas constant, T the absolute temperature, v the molar volume, b the volume excluded by a mole of molecules, and a the parameter representing attractive interactions. Since we are interested only in excluded volume effects, we neglect the attractive interactions. Then, with $a = 0$, we can formally compute the work W required to "blow up" the molecules from point particles to real molecules excluding the volume b:

$$W = -\int_0^b P db = -RT \ln\left(\frac{v-b}{v}\right) \tag{S2}$$

For one molecule, this can be translated into a standard chemical potential difference between a real molecule with excluded volume V_0 and actually occupied volume V_{n} and a point particle occupying the volume V_{n}:

$$\Delta\mu_{\mathrm{st}}^{\mathrm{o}} = -k_{\mathrm{B}}T \ln\left(\frac{V_{\mathrm{n}} - V_0}{V_{\mathrm{n}}}\right) = -k_{\mathrm{B}}T \ln\left(1 - \frac{V_0}{V_{\mathrm{n}}}\right) \tag{S3}$$

where k_{B} is Boltzmann´s constant.

In order to arrive at eq. (32), we consider the motion of detergent head groups to be restricted to the surface area of the micellar core. The steric repulsion then is a function of the ratio of the excluded area (cross-sectional area) of one head group A_{p} and the total surface area of the micellar core per detergent molecule A:

$$\Delta\mu_{\mathrm{st}}^{\mathrm{o}} = -k_{\mathrm{B}}T \ln\left(1 - \frac{A_{\mathrm{p}}}{A}\right) \tag{S4}$$

Dividing by $k_{\mathrm{B}}T$, we obtain g_{st}.

S2: Alternative derivation of eq. (20)

Phillips investigated the formation of micelles for the case of ionic detergents, where the micelles bear an effective charge p and are interacting with counter ions.[14] We repeat his derivation for nonionic detergents with $p = 0$ and no counter ions. With the assumption of a fixed aggregation number m (mass action model), we can write the equilibrium constant K for micelle formation as

$$K = \frac{z}{my^m} = e^{-mg_{\text{mic}}} \tag{S5}$$

where z/m is the mole fraction of micelles, y the mole fraction of detergent monomers, and g_{mic} the micellization free energy per detergent molecule in the micelle divided by the thermal energy $k_{\text{B}}T$. Then,

$$z = mKy^m \tag{S6}$$

Phillips assumes ϕ to be an ideal property, i. e., a measurable quantity that is proportional to the mole fraction (or concentration) of detergent monomers or micelles, so that the breaking point in the experimental titration curve coincides with the breaking point in the dependence of y or z/m on the total detergent concentration x. Corresponding to his equations (5) and (6), we have in our symbolism

$$\phi = A_1 y + A_2 \frac{z}{m} = A_1 y + A_2 K y^m \tag{S7}$$

(with A_1 and A_2 being proportionality constants) as well as

$$x = y + z = y + Kmy^m \tag{S8}$$

Differentiating both equations (S7) and (S8) with respect to y, we find

$$\frac{d\phi}{dy} = A_1 + A_2 Kmy^{m-1} \tag{S9}$$

$$\frac{dx}{dy} = 1 + Km^2 y^{m-1} \tag{S10}$$

Then,

$$\frac{d\phi}{dx} = \frac{d\phi}{dy}\frac{dy}{dx} = \frac{A_1 + A_2 Kmy^{m-1}}{1 + Km^2 y^{m-1}} \tag{S11}$$

$$\frac{d^2\phi}{dx^2} = \frac{dy}{dx}\frac{d}{dy}\left(\frac{d\phi}{dx}\right) = K\frac{[A_2 m - A_1 m^2](m-1)my^{m-2}}{(1 + Km^2 y^{m-1})^3} \tag{S12}$$

and

$$\frac{d^3\phi}{dx^3} = \frac{dy}{dx}\frac{d}{dy}\left(\frac{d^2\phi}{dx^2}\right) = K\frac{[A_2 m - A_1 m^2]}{(1 + Km^2 y^{m-1})^5}\Omega \tag{S13}$$

with

$$\Omega = (m-2)(m-1)y^{m-3} + Km^2(m-1)(1-2m)y^{2m-4} \tag{S14}$$

Assuming $A_2 m - A_1 m^2 \neq 0$, eq. (16) of the main text implies $\Omega = 0$. Dividing this result by $m - 1$ and by y^{m-3} yields

$$0 = m - 2 + Km^2(1-2m)y^{m-1} \tag{S15}$$

It follows from eq. (S8) that

$$y^m = \frac{x - y}{Km} \tag{S16}$$

Plugging eq. (S16) into (S15) and rearranging yields eq. (20) of the main text.

S3: Draft of a non-ideal solution model

In the draft model, the Gibbs free energy of the solution is given by

$$G = G_{\mathrm{f}} + G_{\mathrm{mix}} + G_{\mathrm{int}} \tag{S17}$$

where G_{f} is given by eq. (3) of the main text. The difference to the ideal solution model is in the free energy of mixing, G_{mix}, which we want to improve by considering excluded volume effects, and in the free energy of interaction between solutes, G_{int}, which we shall describe in the framework of a mean field approach.

The root idea of considering excluded volume effects in the entropy of mixing as formulated by Hildebrand[15] is based on an equation of state of the van-der-Waals type, where as in the modelling of the steric free energy g_{st} (see Text S1 above), $a = 0$. Then, the entropy change of expanding the gas isothermally from a volume V_1 to a volume V_2 is given by

$$\Delta S = R \ln\left(\frac{V_2 - b}{V_1 - b}\right) \tag{S18}$$

Thus, the entropy change is determined by the change in *free volume* $V - b$.

In the following we work again with particle numbers and the Boltzmann constant instead of mole numbers and the gas constant. To obtain the entropy of mixing for a binary liquid mixture, we consider the transfer from N_1 molecules of type 1 and N_2 molecules of type 2 with excluded volumes b_1 and b_2, respectively, from the pure state, where they occupy the respective volumes v_1 and v_2, to a solution with total particle number $N_1 + N_2$. Since the considered molecules are polyatomic, they have internal degrees of freedom (DOFs) due to vibrations, rotations, and librations. We follow Hildebrand[15] and assume that the energy in these internal DOFs is not significantly different in the pure state and in the solution. Then, the entropy of mixing is obtained by considering for each component the expansion from its free volume $N_i(\mathrm{v}_i - b_i)$ in the pure state to its free volume $V - N_1 b_1 - N_2 b_2$ in the mixture, where V is the actual volume of the solution:

$$\Delta S = k_{\mathrm{B}}\left[N_1 \ln\left\{\frac{V - N_1 b_1 - N_2 b_2}{N_1(\mathrm{v}_1 - b_1)}\right\} + N_2 \ln\left\{\frac{V - N_1 b_1 - N_2 b_2}{N_2(\mathrm{v}_2 - b_2)}\right\}\right] \tag{S19}$$

A simplifying assumption is that the solution is *additive*,[15] i. e., there is no volume change due to mixing:

$$V = N_1 \mathrm{v}_1 + N_2 \mathrm{v}_2 \tag{S20}$$

Then, eq. (S19) becomes

$$\frac{\Delta S}{k_{\mathrm{B}}} = N_1 \ln\left\{\frac{N_1(\mathrm{v}_1 - b_1) + N_2(\mathrm{v}_2 - b_2)}{N_1(\mathrm{v}_1 - b_1)}\right\} + N_2 \ln\left\{\frac{N_1(\mathrm{v}_1 - b_1) + N_2(\mathrm{v}_2 - b_2)}{N_2(\mathrm{v}_2 - b_2)}\right\} \tag{S21}$$

With these settings, we now consider an aqueous solution of a detergent with – for the sake of simplicity – only one type of co-solute. We shall use the index "w" for water, "c" for the co-solute, and "ν" for a detergent aggregate with ν detergent molecules. We introduce the abbreviations

$$\omega_i = \mathrm{v}_i - b_i \tag{S22}$$

and

$$\varpi = N_{\mathrm{w}}\omega_{\mathrm{w}} + N_{\mathrm{c}}\omega_{\mathrm{c}} + \sum_{\nu} N_{\nu}\omega_{\nu} \tag{S23}$$

so that the free energy of mixing becomes

$$G_{\mathrm{mix}} = -k_{\mathrm{B}}T\left[N_{\mathrm{w}} \ln\left\{\frac{\varpi}{N_{\mathrm{w}}\omega_{\mathrm{w}}}\right\} + N_{\mathrm{c}} \ln\left\{\frac{\varpi}{N_{\mathrm{c}}\omega_{\mathrm{c}}}\right\} + \sum_{\nu} N_{\nu} \ln\left\{\frac{\varpi}{N_{\nu}\omega_{\nu}}\right\}\right] \tag{S24}$$

To model G_{int}, we employ the Bragg-Williams approximation:

$$G_{\text{int}} = k_{\text{B}}T\left[J_{\text{c}}\frac{N_c^2}{\bar{N}} + \sum_{\nu}\left(J_{\text{c}\nu}\frac{N_{\text{c}}N_\nu}{\bar{N}} + J_\nu\frac{N_\nu^2}{\bar{N}}\right) + \sum_{\substack{\nu\nu' \\ \nu'>\nu}} J_{\nu\nu'}\frac{N_\nu N_{\nu'}}{\bar{N}}\right] \tag{S25}$$

Here, we only consider interactions between solutes, and J_i is the *exchange parameter* of the respective interaction.[16] Note that the total particle number

$$\bar{N} = N_{\text{w}} + N_{\text{c}} + \sum_{\nu} N_\nu \tag{S26}$$

is different from N_{tot} defined in the main text, as N_ν counts the micelles rather than the detergent molecules in micelles.

Eq. (9) requires the computation of the partial derivative

$$\frac{\partial G}{\partial N_\nu} = \frac{\partial G_{\text{f}}}{\partial N_\nu} + \frac{\partial G_{\text{mix}}}{\partial N_\nu} + \frac{\partial G_{\text{int}}}{\partial N_\nu} \tag{S27}$$

We have

$$\frac{\partial G_{\text{f}}}{\partial N_\nu} = \nu\mu_\nu^{\text{o}} \tag{S28}$$

$$\begin{aligned}
\frac{\partial G_{\text{mix}}}{\partial N_\nu} &= -k_{\text{B}}T\Bigg[N_{\text{w}}\frac{N_{\text{w}}\omega_{\text{w}}}{\varpi}\frac{\omega_\nu}{N_{\text{w}}\omega_{\text{w}}} + N_{\text{c}}\frac{N_{\text{c}}\omega_{\text{c}}}{\varpi}\frac{\omega_\nu}{N_{\text{c}}\omega_{\text{c}}} + \sum_{\substack{\nu' \\ \nu'\neq\nu}} N_{\nu'}\frac{N_{\nu'}\omega_{\nu'}}{\varpi}\frac{\omega_\nu}{N_{\nu'}\omega_{\nu'}} \\
&\qquad + \ln\left\{\frac{\varpi}{N_\nu\omega_\nu}\right\} + N_\nu\frac{N_\nu\omega_\nu}{\varpi}\left(\frac{\omega_\nu}{N_\nu\omega_\nu} - \frac{\varpi}{N_\nu^2\omega_\nu}\right)\Bigg] \\
&= -k_{\text{B}}T\left[\left(N_{\text{w}} + N_{\text{c}} + \sum_{\substack{\nu' \\ \nu'\neq\nu}} N_{\nu'}\right)\frac{\omega_\nu}{\varpi} + \ln\left\{\frac{\varpi}{N_\nu\omega_\nu}\right\} + \frac{N_\nu}{\varpi}\left(\omega_\nu - \frac{\varpi}{N_\nu}\right)\right] \\
&= -k_{\text{B}}T\left[\bar{N}\frac{\omega_\nu}{\varpi} - \ln\left\{\frac{N_\nu\omega_\nu}{\varpi}\right\} - 1\right] = k_{\text{B}}T\left[1 + \ln\left\{\frac{N_\nu\omega_\nu}{\varpi}\right\} - \bar{N}\frac{\omega_\nu}{\varpi}\right]
\end{aligned} \tag{S29}$$

and

$$\begin{aligned}
\frac{\partial G_{\text{int}}}{\partial N_\nu} &= k_{\text{B}}T\Bigg[-J_{\text{c}}\frac{N_c^2}{\bar{N}^2} - \sum_{\nu'}\left(J_{\text{c}\nu'}\frac{N_{\text{c}}N_{\nu'}}{\bar{N}^2} + J_{\nu'}\frac{N_{\nu'}^2}{\bar{N}^2}\right) + J_{\text{c}\nu}\frac{N_{\text{c}}}{\bar{N}} + 2J_\nu\frac{N_\nu}{\bar{N}} \\
&\qquad - \sum_{\substack{\nu'\nu'' \\ \nu''>\nu'}} J_{\nu'\nu''}\frac{N_{\nu'}N_{\nu''}}{\bar{N}^2} + \sum_{\substack{\nu' \\ \nu'\neq\nu}} J_{\nu\nu'}\frac{N_{\nu'}}{\bar{N}}\Bigg] \\
&= k_{\text{B}}T\Bigg[\frac{1}{\bar{N}}\left(J_{\text{c}\nu}N_{\text{c}} + 2J_\nu N_\nu + \sum_{\substack{\nu' \\ \nu'\neq\nu}} J_{\nu\nu'}N_{\nu'}\right) \\
&\qquad - \frac{1}{\bar{N}^2}\left(J_{\text{c}}N_c^2 + \sum_{\nu'}\left(J_{\text{c}\nu'}N_{\text{c}}N_{\nu'} + J_{\nu'}N_{\nu'}^2\right) + \sum_{\substack{\nu'\nu'' \\ \nu''>\nu'}} J_{\nu'\nu''}N_{\nu'}N_{\nu''}\right)\Bigg]
\end{aligned} \tag{S30}$$

These equations are still quite general. To make progress in understanding, we simplify further by making two approximations:

1) We adopt a mass action model, i. e. we assume that only one type of detergent micelle with aggregation number m exists. This is the same assumption that we made in the main text within the ideal solution model. Thus, we have only $\nu = 1$ and $\nu = m$.

2) We neglect interactions between micelles, between detergent monomers as well as between detergent monomers and micelles. Note that G_{int} does not account for micelle formation, but only for the interaction between separated solutes. Thus, we consider only such interactions between the co-solute and detergent monomers as well as micelles and between co-solute molecules.

With these approximations, we obtain from eq. (9) together with eqs. (S27) to (S30):

$$\mu_1^{\text{o}} + k_{\text{B}}T\left[1 + \ln\left\{\frac{N_1\omega_1}{\varpi}\right\} - \overline{N}\frac{\omega_1}{\varpi} + \frac{J_{\text{c}1}N_{\text{c}}}{\overline{N}} - \frac{1}{\overline{N}^2}\left(J_{\text{c}}N_c^2 + J_{\text{c}1}N_{\text{c}}N_1 + J_{\text{c}m}N_{\text{c}}N_m\right)\right] = \mu_m^{\text{o}}$$
$$+\frac{k_{\text{B}}T}{m}\left[1 + \ln\left\{\frac{N_m\omega_m}{\varpi}\right\} - \overline{N}\frac{\omega_m}{\varpi} + \frac{J_{\text{c}m}N_{\text{c}}}{\overline{N}} - \frac{1}{\overline{N}^2}\left(J_{\text{c}}N_c^2 + J_{\text{c}1}N_{\text{c}}N_1 + J_{\text{c}m}N_{\text{c}}N_m\right)\right] \quad \text{(S31)}$$

Eq. (S31) can be rearranged to give

$$-\frac{m(\mu_m^{\text{o}} - \mu_1^{\text{o}})}{k_{\text{B}}T} = \ln\left\{\frac{N_m\omega_m}{(N_1\omega_1)^m}\varpi^{m-1}\right\} + \frac{\overline{N}}{\varpi}(m\omega_1 - \omega_m) + \frac{1}{\overline{N}}\left(J_{\text{c}m}N_{\text{c}} - mJ_{\text{c}1}N_{\text{c}}\right)$$
$$+\frac{1}{\overline{N}^2}(m-1)\left(J_{\text{c}}N_c^2 + J_{\text{c}1}N_{\text{c}}N_1 + J_{\text{c}m}N_{\text{c}}N_m - \overline{N}^2\right) \quad \text{(S32)}$$

This equation can be further simplified by assuming that detergent and co-solute are diluted enough so that terms in the order of $N_i^2/\overline{N}^2$ can be neglected. Considering the definition in eq. (12), we thus obtain:

$$-mg_{\text{mic}} = \ln\left\{\frac{N_m\omega_m}{(N_1\omega_1)^m}\varpi^{m-1}\right\} + \frac{\overline{N}}{\varpi}(m\omega_1 - \omega_m) + (1-m) + \frac{N_{\text{c}}}{\overline{N}}\left(J_{\text{c}m} - mJ_{\text{c}1}\right) \quad \text{(S33)}$$

The first three terms on the right-hand side of eq. (S33) originate from the consideration of excluded volume effects in the entropy of mixing, while the last term is due to interactions between detergent and co-solute. Note that the latter interaction does not include an attachment of co-solute molecules to either detergent monomers or micelles. Further, eq. (S33) is not a model for g_{mic}, but rather describes the relation of g_{mic} to the various particle numbers as does eq. (13) of the main text in the ideal solution model.

To find the connection to the mole fractions X_i defined in the main text, we make another simplifying assumption discussed by Hildebrand:[15] We assume that the free volumes ω_i are proportional to the molar volumes v_i with a species-independent proportionality constant. (Hildebrand actually discussed the molal volumes.[15]) This approximation implies that the volume fraction Y_i of species i (with mole number n_i and molarity c_i) is given by

$$Y_i = \frac{N_i\omega_i}{\varpi} = \frac{N_i\text{v}_i}{V} = \frac{n_iv_i}{V} = c_iv_i = c_{\text{tot}}v_iX_i \quad \text{(S34)}$$

Recall that for micelles, we defined X_ν as the mole fraction of detergent in micelles rather than as the mole fraction of micelles. Thus, eq. (6) of the main text implies, that in the particular case $i = m$, we have

$$Y_m = c_{\text{tot}}v_m\frac{X_m}{m} \quad \text{(S35)}$$

Here, Y_m and v_m are the volume fraction and molar volume, respectively, of micelles with aggregation number m. Eq. (S34) also implies that

$$\frac{\omega_i}{\varpi} = \frac{\text{v}_i}{V} = \frac{v_i}{N_{\text{A}}V} \quad \text{(S36)}$$

where N_{A} is Avogadro´s number, so that

$$\bar{N}\frac{\omega_i}{\varpi} = \frac{\bar{N}v_i}{N_A V} \approx c_{\text{tot}}v_i \tag{S37}$$

Here, we neglected the contribution of detergent to $\bar{N}$ in the last step, so that $\bar{N} \approx N_{\text{tot}}$ and $c_{\text{tot}} \approx \bar{N}/(N_A V)$. Note that this approximation was already applied in Section 2.6, where the contribution of detergent to $c_{\text{tot}} \approx N_{\text{tot}}/(N_A V)$ was neglected. Then,

$$\frac{\bar{N}}{\varpi}(m\omega_1 - \omega_m) \approx c_{\text{tot}}(mv_1 - v_m) = mc_{\text{tot}}\xi \tag{S38}$$

To have a short notation, we introduced the quantity

$$\xi = \frac{1}{m}(mv_1 - v_m) = v_1 - \frac{v_m}{m} \tag{S39}$$

which represents the volume difference between m detergent monomers in solution and a micelle with aggregation number m per detergent molecule in the micelle. It may be argued that this quantity is practically zero, but $\xi = 0$ would be an approximation. Further, with the neglect of detergent contributions to $\bar{N}$, we obtain for the co-solute $N_c/\bar{N} \approx X_c$. Similar to eq. (S39), we introduce the quantity

$$\zeta = \frac{1}{m}(J_{cm} - mJ_{c1}) = \frac{J_{cm}}{m} - J_{c1} \tag{S40}$$

representing the difference in interaction of the co-solute with a micelle with aggregation number m and with m detergent monomers per detergent molecule in the micelle. Then, eq. (S33) becomes:

$$\begin{aligned} -mg_{\text{mic}} &= \ln\left\{\frac{Y_m}{Y_1^m}\right\} + (1-m) + m(c_{\text{tot}}\xi + X_c\zeta) \\ &= \ln\left\{\frac{X_m}{mX_1^m}\right\} + \ln\left\{\frac{c_{\text{tot}}v_m}{(c_{\text{tot}}v_1)^m}\right\} + (1-m) + m(c_{\text{tot}}\xi + X_c\zeta) \end{aligned} \tag{S41}$$

We now see clearly that with respect to the ideal solution model represented by eq. (13), we have three additional terms. While $(1-m)$ is known, the other two of these additional terms remain to be quantified.

If $\xi \approx 0$, we may use $v_m \approx mv_1$ and compute v_m according to

$$v_m = N_A \mathrm{v}_m = N_A \frac{4}{3}\pi(a+d)(b+d)^2 \tag{S42}$$

where v_m is now approximated by the volume of a micelle with the parameters a and b describing the spheroidal core and d being the thickness of the head group layer (for the volume of an oblate spheroid, see eq. (16) in ref. 1). Values for v_m,

$$\Psi = \ln\left\{\frac{c_{\text{tot}}v_m}{(c_{\text{tot}}v_1)^m}\right\} = \ln\left\{\frac{c_{\text{tot}}v_m m^m}{(c_{\text{tot}}v_m)^m}\right\} = (1-m)\ln\{c_{\text{tot}}v_m\} + m\ln m \tag{S43}$$

and

$$\psi = \frac{1}{m}(\Psi + 1 - m) = \frac{\Psi}{m} + \frac{1}{m} - 1 \tag{S44}$$

are compiled in Table 6.

It follows that in the non-ideal solution model, eq. (13) of the main text is replaced by

$$X_m = mX_1^m e^{-m(g_{\text{mic}} + \psi + c_{\text{tot}}\xi + X_c\zeta)} \tag{S45}$$

with the various contributions to non-ideal behavior added to g_{mic}, while eq. (21) becomes

$$g_{\text{mic}} = -\psi - c_{\text{tot}}\xi - X_c\zeta + \frac{m-1}{m}\ln X_{\text{CMC}} + \frac{1}{m}\ln\left\{\frac{m(2m^2-m)^m}{(m-2)(2m^2-2)^{m-1}}\right\} \tag{S46}$$

Table 6: Computation of the quantity ψ defined in eq. (S44) for alkyl maltosides with n carbon atoms in the alkyl chain, using eq. (S42) with $d = 6.15$ Å.[6]

n	8	9	10	11	12
a / Å	10.45	11.35	12.25	13.15	14.05
b / Å	18.25	20.88	23.50	26.13	28.75
$(a + d)$ / Å	16.60	17.50	18.40	19.30	20.20
$(b + d)$ / Å	24.40	27.03	29.65	32.28	34.90
m	47	62	85	106	140
$m \ln m$	180.96	255.88	377.63	494.32	691.83
v_m /M^{-1}	24.93	32.25	40.80	50.73	62.06
$c_{\mathrm{tot}} v_m$ for $c_{\mathrm{tot}} = 54.65$ M	1362.42	1762.46	2229.72	2772.39	3391.58
$c_{\mathrm{tot}} v_m$ for $c_{\mathrm{tot}} = 55.32$ M	1379.13	1784.07	2257.06	2806.38	3433.16
Ψ for $c_{\mathrm{tot}} = 54.65$ M	–151.02	–200.06	–269.98	–338.06	–438.11
Ψ for $c_{\mathrm{tot}} = 55.32$ M	–151.58	–200.81	–271.00	–339.34	–439.80
ψ for $c_{\mathrm{tot}} = 54.65$ M	–4.19	–4.21	–4.16	–4.18	–4.12
ψ for $c_{\mathrm{tot}} = 55.32$ M	–4.20	–4.22	–4.18	–4.19	–4.13

ESI References

1. F. Müh, D. DiFiore and A. Zouni, *Phys. Chem. Chem. Phys.*, 2015, **17**, 11678-11691.
2. K. A. Sharp, A. Nicholls, R. F. Fine and B. Honig, *Science*, 1991, **252**, 106-109.
3. A. Bothe, Master thesis, Humboldt Universität zu Berlin, 2021.
4. R. C. Oliver, J. Lipfert, D. A. Fox, R. H. Lo, S. Doniach and L. Columbus, *Plos One*, 2013, **8**, e62488.
5. E. R. S. Kunji, M. Harding, P. J. G. Butler and P. Akamine, *Methods*, 2008, **46**, 62-72.
6. J. Lipfert, L. Columbus, V. B. Chu, S. A. Lesley and S. Doniach, *J. Phys. Chem. B*, 2007, **111**, 12427-12438.
7. P. J. Tummino and A. Gafni, *Biophys J*, 1993, **64**, 1580-1587.
8. T. Jumpertz, B. Tschapek, N. Infed, S. H. J. Smits, R. Ernst and L. Schmitt, *Anal. Biochem.*, 2011, **408**, 64-70.
9. H. Alpes, K. Allmann, H. Plattner, J. Reichert, R. Riek and S. Schulz, *Biochim. Biophys. Acta*, 1986, **862**, 294-302.
10. W. J. De Grip and P. H. M. Bovee-Geurts, *Chem. Phys. Lipids*, 1979, **23**, 321-335.
11. M. F. Sanner, A. J. Olson and J. C. Spehner, *Biopolymers*, 1996, **38**, 305-320.
12. R. Nagarajan and E. Ruckenstein, *Langmuir*, 1991, **7**, 2934-2969.
13. R. Nagarajan, *Adv. Colloid Interface Sci.*, 1986, **26**, 205-264.
14. J. N. Phillips, *Trans. Faraday Soc.*, 1955, **51**, 561-569.
15. J. H. Hildebrand, *J. Chem. Phys.*, 1947, **15**, 225-228.
16. K. A. Dill and S. Bromberg, *Molecular Driving Forces*, Garland Science, London, New York, 2011.
